# High-Resolution Mapping of Spontaneous Mitotic Recombination Hotspots on the 1.1 Mb Arm of Yeast Chromosome IV

**DOI:** 10.1371/journal.pgen.1003434

**Published:** 2013-04-04

**Authors:** Jordan St. Charles, Thomas D. Petes

**Affiliations:** 1 Department of Molecular Genetics and Microbiology, Duke University Medical Center, Durham, North Carolina, United States of America; 2 Department of Pharmacology and Cancer Biology, Duke University Medical Center, Durham, North Carolina, United States of America; NYU Langone Medical Center, United States of America

## Abstract

Although homologous recombination is an important pathway for the repair of double-stranded DNA breaks in mitotically dividing eukaryotic cells, these events can also have negative consequences, such as loss of heterozygosity (LOH) of deleterious mutations. We mapped about 140 spontaneous reciprocal crossovers on the right arm of the yeast chromosome IV using single-nucleotide-polymorphism (SNP) microarrays. Our mapping and subsequent experiments demonstrate that inverted repeats of Ty retrotransposable elements are mitotic recombination hotspots. We found that the mitotic recombination maps on the two homologs were substantially different and were unrelated to meiotic recombination maps. Additionally, about 70% of the DNA lesions that result in LOH are likely generated during G1 of the cell cycle and repaired during S or G2. We also show that different genetic elements are associated with reciprocal crossover conversion tracts depending on the cell cycle timing of the initiating DSB.

## Author Summary

Double-strand breaks (DSBs) are DNA lesions that can be fatal to a cell if left unrepaired. They can be caused by exogenous sources, such as gamma radiation, or endogenous stresses, such as high levels of transcription. Yeast cells primarily repair DSBs that are initiated outside of meiosis by mitotic recombination, which can result in physical exchanges between chromosomes, known as crossovers. We created a mitotic recombination map of one chromosome arm, representing 10% of the genome. This recombination map allows us to determine which regions of the chromosome arm are more susceptible to DNA damage than other regions. We were able to determine that most DSBs that result in detectable genomic changes were initiated prior to DNA replication and that some secondary DNA structures can be recombination hotspots. Recombination can also occur during meiosis, as a method of ensuring proper chromosome segregation. However, previously reported meiotic recombination maps have no correlation with our mitotic recombination map.

## Introduction

A double-stranded break (DSB) is a potentially lethal DNA lesion that can lead to genomic instability and chromosome rearrangements if not promptly repaired. DSBs and other forms of DNA damage (for example, single-stranded nicks or base damage) can result from exogenous (for example, gamma- or UV-radiation) or endogenous sources [1].

Inverted repeats have been called “at-risk motifs” because of their ability to form recombinogenic secondary structures such as hairpins and cruciforms when intrastrand pairing takes place [2]. Hairpin structures can cause replication fork pausing and lead to DSBs [3]. Cruciform structures are thought to promote genome instability because they resemble Holliday junctions that can be cleaved by resolvases. The studies of the effects of inverted repeats on genomic stability have usually been done using repeats introduced into the genome by transformation [4], [5] or in strains with defective DNA replication [6], [7]. Below, we will show that naturally-occurring inverted repeats act as recombination hotspots in strains with unperturbed DNA replication.

**Figure 1:**
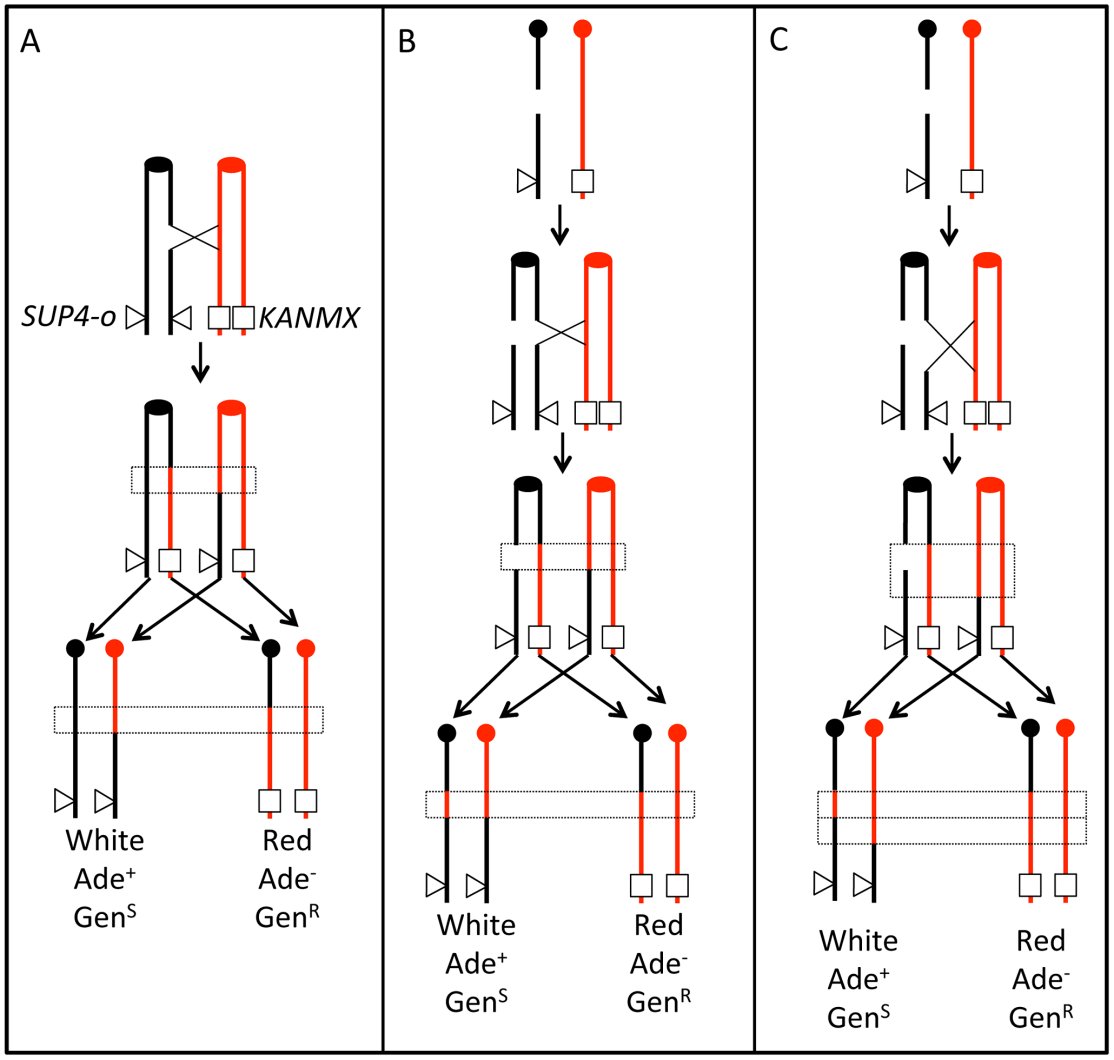
Patterns of gene conversion resulting from the repair of G1- or G2-generated DSBs. Chromatids that are depicted in black represent YJM789-derived chromatids, and those in red represent W303a-derived chromatids. The centromeres are shown as circles. A. 3∶1 conversion event. Repair of a G2-associated DSB results in a 3∶1 gene conversion tracts (enclosed in dotted lines) associated with the LOH event. B. 4∶0 conversion event. A chromosome in a G1 cell is replicated to form two sister chromatids that are broken at approximately the same position. Repair of these two broken chromatids in G2 can result in a region in which all four chromatids have identical SNPs, a 4∶0 conversion tract. In this example, both conversion tracts are of the same length. C. 4∶0/3∶1 hybrid tract. As in Figure 1B, a broken chromosome is replicated to form to two broken sister chromatids. The conversion tracts associated with the repair of the two DSBs, however, are of different lengths, resulting in a 4∶0/3∶1 hybrid tract.

In *S. cerevisiae*, DSBs can be repaired by non-homologous end joining (NHEJ) or by homologous repair (HR) [8]. In HR events, an intact donor DNA molecule (a sister chromatid or a homolog) is used to repair the broken molecule. HR can result in conversions (the non-reciprocal transfer of DNA sequence) and crossovers. Conversion events can be associated or unassociated with reciprocal crossovers (RCOs). Conversion events result from either correction of mismatches in heteroduplex DNA by the mismatch repair system [9], or by repair of a double-stranded DNA gap located near the site of the DSB [10]. Mitotic crossovers often result in LOH distal to the crossover (Figure 1).

Previously, we showed several different types of gene conversion events that likely reflect differences in the timing of the recombination-initiating DSBs in the cell cycle [11], [12]. Figure 1 shows a comparison of conversion tracts resulting from repair of a DSB formed during S/G2 (Figure 1A) or G1 (Figure 1B and 1C); the red and black lines represent homologs with heterozygous SNPs. Figure 1A shows a DSB on a black chromatid during G2 of the cell cycle that is repaired by HR, resulting in a crossover and associated 3∶1 conversion tract (three chromatids with red SNPs and one with black SNPs). Figure 1B shows a DSB on the black homolog during G1 of the cell cycle that is replicated to generate two broken chromatids. Since both chromatids are broken in the same location, only the homolog is available as a template for HR. If one chromatid is repaired resulting in a crossover and associated conversion event, and the second chromatid is repaired as a conversion event unassociated with crossovers, there will be a 4∶0 conversion tract. Figure 1C also shows a DSB on the black homolog during G1 of the cell cycle. Following replication, repair occurs as in Figure 1B, but one broken chromatid has been processed to a greater extent than the other, resulting in unequal-sized conversion tracts. The resulting conversion tract is a 4∶0/3∶1 hybrid tract.

In this study, we map spontaneous crossovers and associated gene conversion events on the right arm of chromosome IV, an interval of about 1.1 Mb, using SNP microarrays to map events at approximately 500 bp resolution. We find that events occur throughout the chromosome arm, although their distribution is non-random. In particular, inverted pairs of transposons at two different locations result in homolog-specific hotspots for crossovers initiated by DSBs during G1. We also demonstrate that LOH events are more frequently a consequence of G1-initiated DSBs than G2-initiated DSBs, and that conversion tracts associated with the G1 DSBs are longer than those associated with G2-initiated DSBs. Finally, we show that different genetic elements are associated with crossovers initiated during G1 and G2. For example, regions of converging replication forks (TER sites; [13]) are associated with G2, but not G1, conversion events.

## Results

### Identification and mapping of crossovers on chromosome IV

We used a colony-color screening assay to identify reciprocal crossovers on chromosome IV, similar to the previously described system [11], [14] (Figure 1A). The diploid JSC25 was generated by mating two sequenced haploid strains, W303a and YJM789, that have 55,000 SNPs relative to each other [15]. We inserted the *KANMX* gene near the telomere on the right arm of the W303a chromosome IV homolog and *SUP4-o* (encoding an ochre-suppressing tRNA) allelically on the YJM789-derived homolog. The *KANMX* gene renders the cell resistant to geneticin (Gen^R^), a drug related to the bacteria-specific antibiotic kanamycin. JSC25 is also homozygous for the ochre-suppressible *ade2-1* mutation. Diploid strains of this genotype that lack *SUP4-o* form red colonies, strains with one copy of *SUP4-o* form pink colonies, and those with two copies form white colonies. If there is a crossover between the centromere and the *SUP4-o* locus, there is a 50% chance, following chromosome segregation that the two daughter cells will be homozygous for the region centromere-distal to the crossover. If the crossover occurs at the time that the diploid JSC25 is plated, a red/white sectored colony will be formed (Figure 1A), in which the white side of the sector is geneticin-sensitive due to loss of the *KANMX* gene, and the red side of the sector is Ade^−^ due to loss of *SUP4-O*. The frequency of red/white sectored colonies (3.1×10^−5^) in JSC25 is reflective of the rate of crossovers between *CEN4* and the *KANMX/SUP4-o* markers. Since only one half of reciprocal crossovers result in a sectored colony, the rate of crossovers on the right arm of chromosome IV is 6.2×10^−5^/division.

We mapped crossovers in about 140 sectored colonies of JSC25. We monitored about 2300 SNPs between *CEN4* and the *KANMX/SUP4-o* loci within the 1.1 Mb interval. As in our previous study [16], for each SNP, we designed four 25-base oligonucleotides: one for each Watson and Crick strand of each homolog. The normalized ratio of experimental to reference hybridization of these samples to each oligonucleotide allowed us to determine whether the experimental strains were heterozygous or homozygous for a specific SNP.

**Figure 2:**
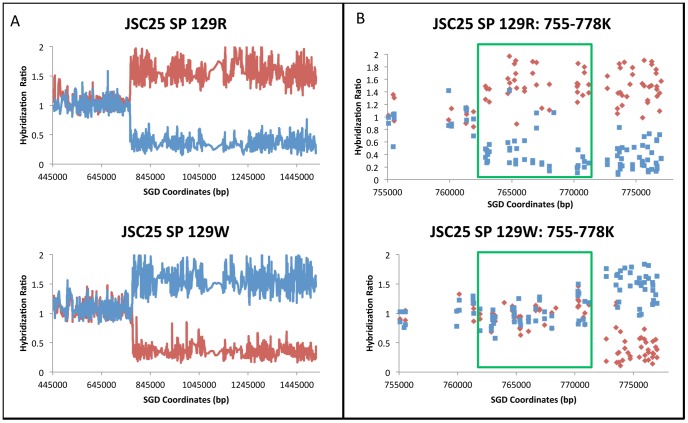
Mapping of a crossover with an associated 3∶1 conversion event by SNP microarrays. The values on the Y-axis show the experimental/reference hybridization ratio of genomic DNA to oligonucleotides that are specific to SNPs from the W303a and YJM789 backgrounds. The values on the X-axis indicate the SGD coordinates of the SNPs along chromosome IV. The red and blue lines or data points depict the hybridization levels of probes specific for the W303a and YJM789 homologs, respectively. Where both the red and blue lines have a value near 1, the diploid is heterozygous for the SNPs. If the strain is homozygous for the W303a variant, the red signal increases to about 1.6 and the blue signal diminishes to about 0.3. If the strain is homozygous for the YJM789 variant, the reverse pattern is observed. A. Low-resolution depiction of a reciprocal crossover analyzed by the SNP microarrays. The top and bottom plots represent the red and white sectors, respectively. In both the red and white sectors, there is a transition between heterozygosity and homozygosity at approximately SGD coordinate 770 kb. B. High-resolution depiction of a reciprocal crossover. Each data point indicates the hybridization level to a specific oligonucleotide. Each square and diamond indicates the hybridization level to a specific oligonucleotide. The genomic DNA from the red sector has a transition between heterozygosity and homozygosity at approximately coordinate 762.5 kb (top panel), whereas genomic DNA from the white sector has a transition at about coordinate 772 kb. The regions shown in green rectangles represent the 3∶1 gene conversion event.

Figure 2 shows an example of the analysis of genomic DNA from a sectored colony (JSC25 SP 129). The hybridization of genomic DNA to the W303a- and YJM789-derived SNPs is shown by red and blue lines, respectively. In the low-resolution analysis (Figure 2A), genomic DNA hybridizes equally efficiently to both W303a- and YJM789-derived SNPs (normalized ratio of 1) from SGD coordinates 445 kb (the location of *CEN4*) to 770 kb. Near coordinate 770 kb in Figure 2A, there is a transition from heterozygosity to homozygosity for the W303a and YJM789 forms of the SNPs in the red (top) and white (bottom) sectors, respectively. Based on the higher resolution depiction (Figure 2B), it is clear that the transition points between heterozygosity and homozygosity are different between the two sides of the sector, occurring near 762 kb and 772 kb in the red and white sectors, respectively. The boxed region in Figure 2B represents a 3∶1 gene conversion tract (Figure 1A). This pattern indicates that the gene conversion tract was initiated by a DNA lesion on the YJM789-derived chromosome, since the chromosome with the lesion acts as a recipient during the conversion event [17]. Microarray data illustrating a hybrid conversion tract are shown in Figure S1.

Of 139 crossovers analyzed, 121 (87%) were associated with a contiguous gene conversion event. Of the 121 conversion tracts, there were 29 simple 3∶1 conversions, 7 simple 4∶0 conversions, and 46 simple 4∶0/3∶1 or 3∶1/4∶0/3∶1 hybrid conversions; 39 tracts had more complicated patterns of conversion (Text S1). Summaries of various features of all conversion tracts observed in our study are presented in Tables S1, S2, S3. No aneuploidy of chromosome IV was observed for any of the sectored colonies. In addition, only one large deletion was detected. In the red sector of colony 42R/W (Class E34 in Table S1), we detected a deletion of about 100 kb located between pairs of inverted Ty elements at SGD coordinates 872 kb and 981 kb. We do not know whether this deletion occurred prior to the crossover or was associated with the crossover.

### Location of crossovers and associated conversions on the right arm of chromosome IV

**Figure 3:**
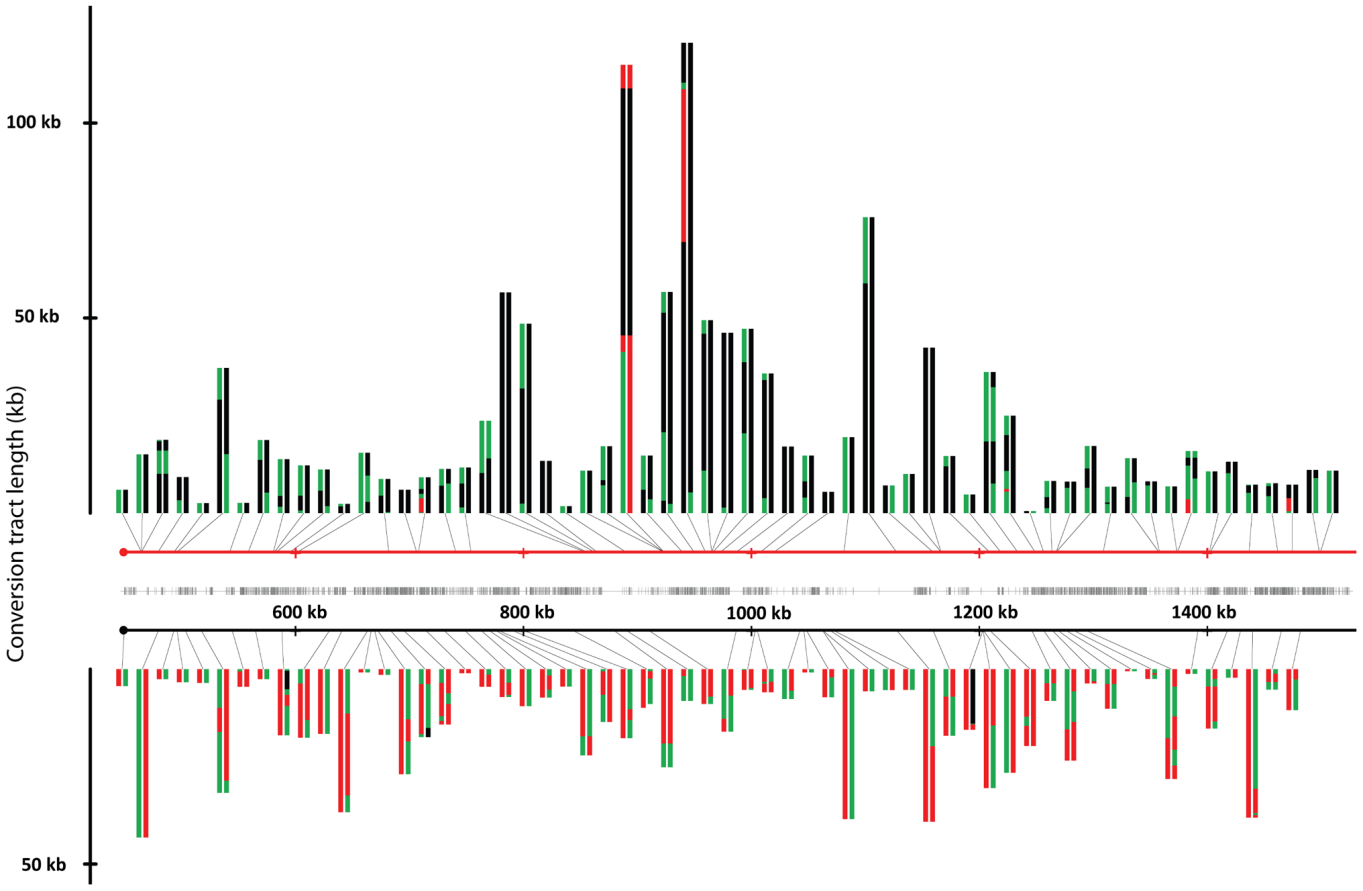
Crossovers and associated gene conversion events in JSC25 along the right arm of chromosome IV. The X-axis represents the SGD coordinates (the centromere is at 449 kb), and the Y-axis represents the length of the individual conversion tract. The red and black horizontal lines in the center of the figure depict the W303a and YJM789 homologs, respectively. Individual conversion tracts are depicted as two parallel vertical lines that are attached to either homolog line by a black connector line which indicates the location of the centromere-proximal margin of the conversion tract. Conversion tracts depicted in the top portion of the figure and connected to the W303a homolog, resulted from DSBs initiated on the W303a homolog. Conversion tracts depicted in the lower half of the figure were initiated by DSBs on the YJM789 homolog. The vertical left and right members of the line pairs show the conversion tract of the red and white sides of the sector, respectively. Green, red, and black show heterozygosity, homozygosity for W303a SNPs, and homozygosity for YJM789 SNPs, respectively. Each segment of the depicted conversion tract is proportional to the size of that segment within the sector. The hash marks between the two homologs indicate the density of the SNPs represented on the microarray.

We collected and analyzed 138 sectors and 139 reciprocal crossovers in the strain JSC25; one sectored strain had a double crossover. The locations of crossovers and associated conversion tracts are shown in Figure 3. The crossovers associated with conversion tracts were separated based on which homolog had the initiating lesion. Events initiated in W303a- or YJM789-derived homologs are presented in the top or bottom of Figure 3, respectively. The conversion tracts are depicted as pairs of lines (discussed in the figure legend) with the lengths of the conversion tracts indicated on the Y-axis. Crossovers unassociated with conversions are not shown in Figure 3, because it is impossible to determine which homolog had the originating DSB. It is apparent that the distribution of conversion events along the chromosome arm is not even. In addition, in certain regions of the chromosome (between SGD coordinates 900 kb and 1000 kb), the conversion tracts appear longer than most of those in other regions of the chromosome.

**Figure 4:**
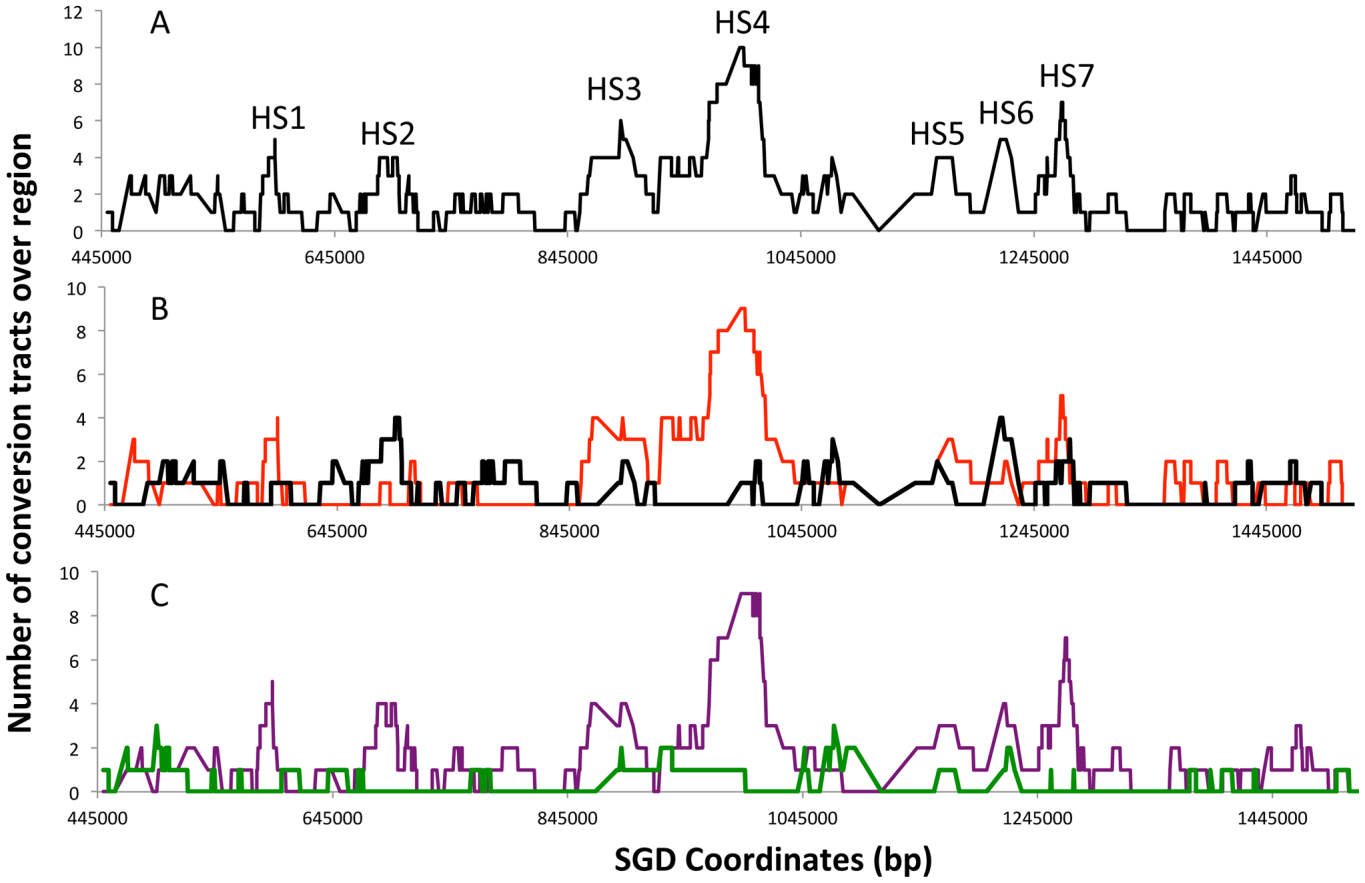
Summary of conversion tracts associated with crossovers along the right arm of chromosome IV. These plots represent the number of times a SNP represented on the microarray is involved in a conversion tract. A SNP was considered involved if it was between the start and end of the conversion tract. A. All conversion tracts. The labels HS1–HS7 represent potential hotspots for recombination. B. Conversion tract distribution of the events initiated on the W303a (red) and YJM789 (black) homolog. C. Conversion tract distribution of events initiated during G1 (purple) or G2 (green line) of the cell cycle.

Figure 4 summarizes the location of crossover-associated gene conversion events in a different way. In this figure, the X-axis shows the SGD coordinates and the Y-axis shows the number of conversion events that include the SNP located at that coordinate summed over all mapped events. The probability of a SNP being included within a conversion tract is a function of both the frequency of nearby DNA lesions and of the lengths of conversion tracts emanating from the initiating DNA lesions. In Figure 4, we label regions where multiple contiguous SNPs were involved multiple times in a gene conversion event as potential hotspots (HS1-HS7). In Figure 4B, we show separately those events initiated on the W303a- and YJM789-derived homologs. These distributions are strikingly different. It is also clear that the distributions of G1-initiated conversions (tracts with 4∶0 segments or other features that indicate a G1-associated DSB) and G2-associated conversions (3∶1 conversions without a 4∶0 segment) are different (Figure 4C). HS3 and HS4 are both G1-specific and specific for the W303a-derived homolog. The mechanism responsible for this specificity will be discussed below.

### Statistical analysis of the distribution of crossovers and conversions

We used several different statistical methods to determine if the distribution of crossovers along the chromosome arm is non-random. In our first statistical analysis, we divided the right arm of chromosome IV into five bins of 200 kb (starting at *CEN4*) and a sixth bin of about 80 kb. We counted the number of crossovers that occurred within each region (additional details discussed in Text S1). If all events are examined, the number of crossovers per bin is approximately proportional to the size of each bin (p = 0.96 by chi-square Goodness-of-Fit). We also analyzed the events separated by the homolog and timing of the initiating lesion (depicted in Figure S2). Both the homolog-specific distributions of crossovers and cell cycle-specific distributions of events were significantly different with p values of 0.01 and 0.007, respectively. The bin from 845–1045 kb (containing HS3 and HS4) had a very significant enrichment of crossovers initiated on the W303a-derived homolog during G1 of the cell cycle compared to a random distribution of this class of event among all of the bins (p = 2.3×10^−5^).

### Comparison of the physical and genetic maps of the right arm of chromosome IV

**Figure 5:**
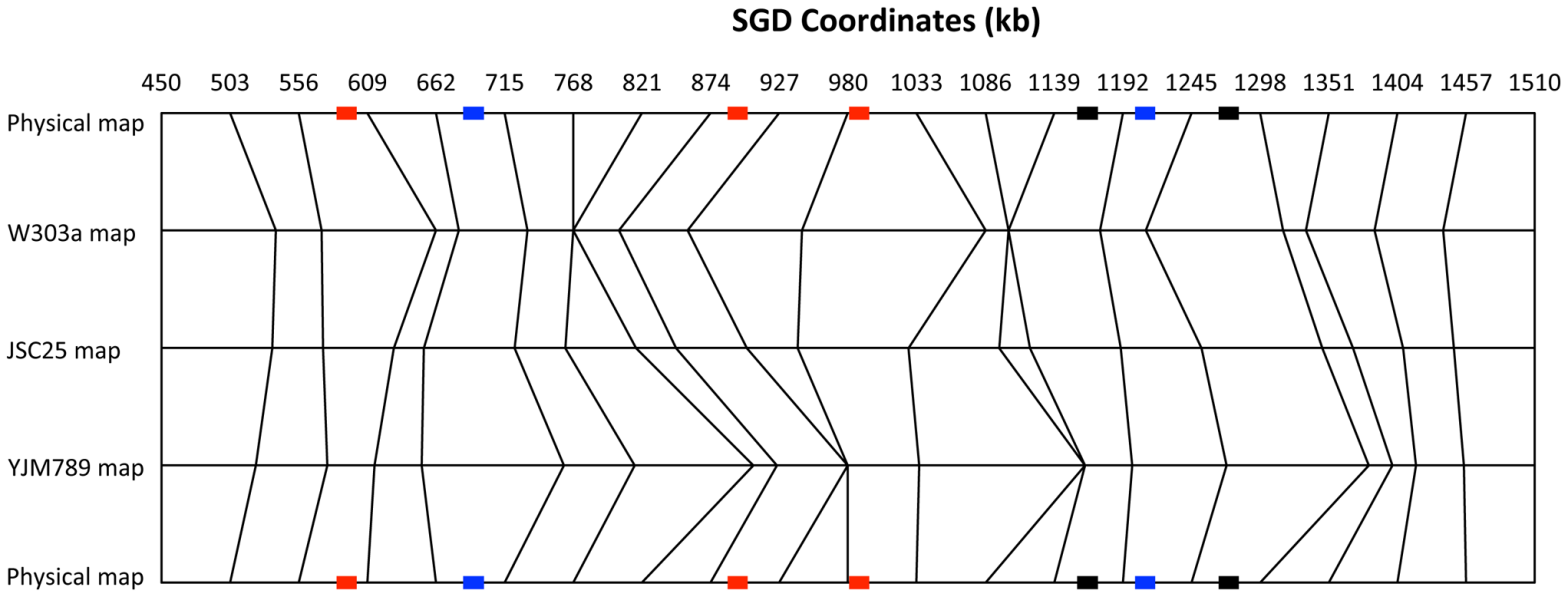
Comparison of physical and genetic mitotic recombination maps. This figure shows a comparison of the physical map of the 1.1 Mb right arm of chromosome IV with a genetic map of mitotic crossovers. We show three genetic maps: all crossovers (JSC25 map), crossovers initiated from the W303a homolog (W303a map), and crossovers initiated from the YJM789 homolog (YJM789 map). The chromosome arm was divided into 20 regions of about 53 kb. The genetic distances are based on the proportion of crossovers within each 53 kb interval, using the “point-analysis” method employed for Figure S2 and described in Text S1. The top and bottom horizontal lines show the physical map. The vertical lines allow comparisons of the genetic maps with the physical maps, and of the different genetic maps with each other. The relative “thinness” and “thickness” of the same segment between the W303a and YJM789 maps indicates whether that region has more crossovers initiated on one homolog in comparison to the other. The recombination hotspots (labeled in Figure 4A) are indicated by rectangles on the physical map lines (top and bottom lines). The red, blue, and black colors indicate hotspots that are specific to the W303a-derived homolog, specific to the YJM789-derived homology, and hotspots that are found on both homologs, respectively.

In Figure 5, we show a comparison between the physical map of the right arm of chromosome IV with the genetic maps based on recombination data from JSC25. In addition, we show separately the data from the W303a- and YJM789-derived homologs. This figure emphasizes the differences in recombination activity between the two homologs. For example, the region between 927 and 980 kb in the map of W303a is larger than expected from the physical map, but there are no events in this segment in the YJM789 map. Conversely, the segment between 768 and 821 kb has no events in the W303a map, but a greater-than-average number of events in the YJM789 map. Such differences are expected because of the differences in the distributions of hotspots on the two homologs. For example, the hotspot activity of HS4 is specific to the W303a chromosome. Thus, we expect an expansion of the genetic map on the W303a-derived homolog near HS4, but no expansion of the map on the YJM789-derived homolog.

We compared the DNA sequences of W303a and YJM789 to determine if any features of the sequence would suggest possible mechanisms for the observed differences in hotspot activity on the two homologs. Although there are many SNPs that distinguish the two homologs, insertions/deletions (in/dels) greater than 50 bp are relatively rare [15], and most of these involve Ty elements or delta elements. These alterations and their approximate position by SGD coordinates are listed below; in this list, boldface indicates that the element is present in W303a and not in YJM789, and the regular typeface indicates that the element is present in YJM789 and not in W303a: 1) delta element at 489 kb, 2) delta element at 513 kb, 3) delta element at 520 kb, 4) Ty element at 645 kb, 5) sigma element at 668 kb, 6) partial Ty1 element at 805 kb, 7) inverted pair of Ty2/Ty1 elements at 872 kb, 8) sigma element at 946 kb, 9) inverted pair of Ty2/Ty1 elements at 981 kb, and 10) unannotated partial delta element at 1151 kb. In addition to retrotransposon-related sequences, there were a small number of other in/dels. The W303a-derived homology has three tandem *ENA* genes located near SGD coordinate 534 kb, whereas the YJM789-derived homolog has only two. The YJM789-derived homolog has a 153 bp insertion in *YDR077W* relative to the same gene on the W303a-derived homolog. The YJM789-derived homolog has a partial deletion of *ARS420* relative to the W303a-derived homolog. Finally, the YJM789 allele of YDR150W has more copies of an internal 192 bp repeat than observed in the W303a allele. Except for these in/dels and numerous SNPs, the genomic sequences of the two homologs are well conserved. For example, no changes were observed for the positions of tRNA genes or G-quadruplex structures.

The significance of these genomic alterations on recombination hotspot activity is unclear with two exceptions. As discussed below, the inverted pairs of Ty elements located at positions 872 kb and 981 kb are likely to be important for the hotspot activities of HS3 and HS4 in the W303a-derived homolog. Other differences in hotspot activity between the two homologs could reflect multiple small changes or homolog-specific alterations in chromatin modifications. In addition, although it is difficult to determine what chromosome elements are associated with most of the hotspots, by analyzing the sequences within all of the conversion tracts, we were able to detect significant associations with certain chromosome elements; this analysis is described below.

Differences in hotspot activities on the two homologs demonstrate the difficulty and, perhaps, the futility of generating a universal mitotic recombination map for *S. cerevisiae*. As shown below, however, differences in the recombination activities of the two homologs can lead to mechanistic insights into mitotic recombination.

### The HS4 hotspot is associated with a pair of inverted Ty elements

The HS4 hotspot, located between coordinates 970 and 1,000 kb, has the highest level of recombination as measured by the number of conversion events (Figure 4A). This hotspot is specific to the W303a-derived homolog (Figure 4B) and is associated with G1-initiated gene conversion events (Figure 4C). Analysis of the DNA sequences in the hotspot region shows that the peak of recombination activity of HS4 overlaps with a closely-spaced inverted pair of Ty elements (*YDRWTy2-3* and *YDRCTy1-3*) located between coordinates 981 and 992 kb. Ty elements are 6 kb retrotransposons present in about 40 copies per haploid genome [18].

To determine whether the hotspot activity of HS4 was related to the inverted pair of Ty elements, we first examined whether both of the haploid parental strains contained the inverted pair of elements. PCR analysis demonstrated that the inverted repeat is in the W303a homolog but not the YJM789 homolog (Figure S3). The observation that the HS4 hotspot is specific to the W303a-derived homolog that contains the inverted pair of Ty elements implicates this structure in the hotspot activity. The spacer between the Ty elements is about 25–66 bp, depending on the degree of mismatches allowed between the repeats (Figure S4).

**Figure 6:**
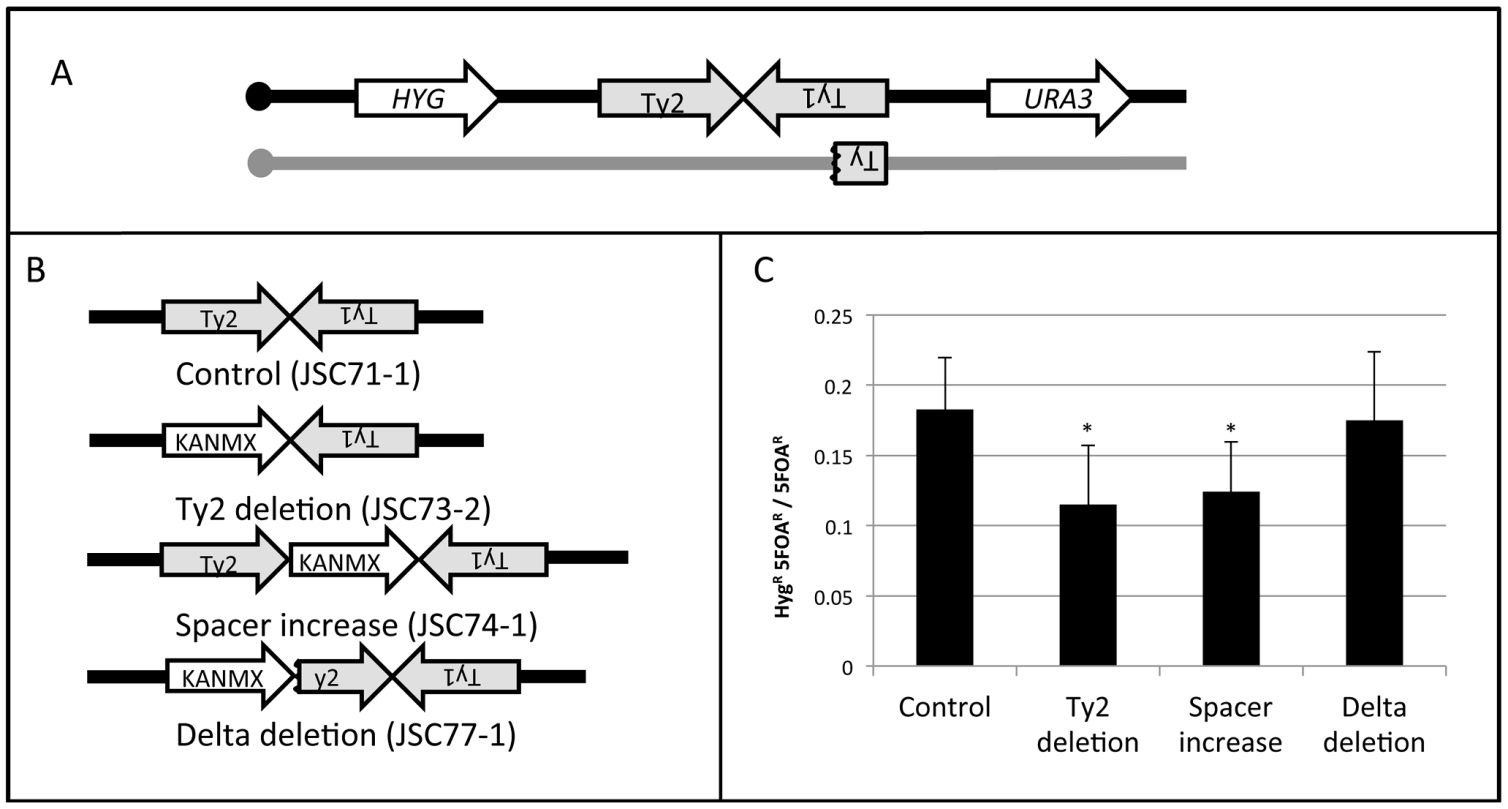
Deletion analysis of the HS4 hotspot. A. Experimental system used to monitor the activity of the wild-type HS4 hotspot and various deletion derivatives. The *URA3* and *HYG* genes were inserted centromere-distal and -proximal to the inverted Ty elements, respectively, on the W303a-derived homolog (black). The YJM789-derived homolog (grey) was unaltered. B. Modifications of the inverted repeat structure. In the control strain JSC71-1, the unmodified hotspot consists of a Ty2 element on the Watson strand, 25 bp of non-Ty sequence, and a Ty1 element on the Crick strand. In the JSC73-2 strain, the Ty2 element is replaced with KANMX. In the JSC74-1 strain, the space between the two Ty elements, is increased by about 2 kb by insertion of the KANMX marker. In the strain JSC77-1, the centromere-proximal delta element of Ty2 was replaced by the KANMX gene. C. Frequency of recombination between *HYG* and *URA3* in the strain with wild-type HS4 activity and in various deletion derivatives. Asterisks indicate a significant (p<0.05) reduction in recombination activity compared to the control strain.

To examine some of the structural features of the HS4 hotspot, we measured hotspot activity in four different isogenic strains (Figure 6) with the wild-type HS4 hotspot (JSC71-1), with a deletion of the HS4-associated Ty2 element (JSC73-2), with an expansion of the spacer between Ty2 and Ty1 (JSC74-1), and with a deletion of one of the delta elements associated with Ty2. For all four strains, we inserted *URA3* about 23 kb centromere-distal to HS4 (1013 kb) and the hygromycin-resistance gene *HYG* about 20 kb centromere-proximal to HS4 (957 kb). These strains are depicted in Figure 6A and 6B.

A crossover initiated at HS4 will result in a daughter cell that is Hyg^R^ and 5-FOA^R^ half of the time. A crossover initiated between *CEN4* and *HYG* will result in a daughter cell that is Hyg^S^ and 5-FOA^R^ half of the time. By monitoring the ratio of Hyg^R^ 5-FOA^R^ recombinants to all 5-FOA^R^ recombinants, we measured the recombination rate between the *HYG* and *URA3* markers. The distance between *CEN4* and *URA3* is about 568 kb and the distance between *HYG* and *URA3* is about 56 kb. Thus, if the numbers of crossovers are proportional to the size of the physical intervals, 10% of the 5-FOA^R^ strains should be Hyg^R^. The observed frequency of derivatives of this class in the strain with the wild-type hotspot (JSC71-1) was 0.17 (Figure 6C). The difference in the number of observed and expected crossovers is statistically significant (p<0.001), as expected since this region contains the HS4 hotspot. In a strain with the deletion of the Ty2 element (JSC73-2) or a strain with an expanded spacer (JSC74-1), the hotspot activity is significantly reduced (Figure 6C). In contrast, in a strain in which the centromere-proximal δ element of Ty2 is deleted (JSC77-1), no significant loss of hotspot activity is observed.

Deletion of the Ty2 element eliminates the possibility of hairpin formation at HS4 and an increase in the spacer between the repeated elements would be expected to substantially reduce the probability of hairpin formation [4], [7]. The 330 bp δ elements flank Ty elements [18] and the 5′ δ element acts as a transcriptional promoter [19]. In the strain containing the deletion of the 5′ δ, Ty2 should no longer be transcriptionally active. The results summarized in Figure 6C, therefore, indicate that the formation of a secondary structure between the two Ty elements is necessary, but the transcriptional activities of both of the Ty elements are not required for hotspot activity at HS4.

The HS3 hotspot (Figure 4) also maps at the positions of an inverted pair of Ty elements (*YDRWTy2-2* and *YDRCTy1-2*) separated by a spacer of about 100 bp. As with HS4, this hotspot is specific to the W303a-derived homolog and G1-specific. Since the YJM789-derived homolog does not have this inverted pair of Ty elements [15], we assume that the hotspot activity of HS3 is a consequence of formation of a secondary structure.

### Types of gene conversion events: Inferences about the timing of the recombinogenic DSBs during the cell cycle

Most (121/139; 87%) of the crossovers examined in our study were associated with gene conversion. Of these conversion tracts, about two-thirds (82/121) are simple 3∶1 conversions, simple 4∶0 conversions, or hybrid 3∶1/4∶0 or 3∶1/4/0/3∶1 tracts as diagrammed in Figure 1. As discussed previously, the simple 3∶1 tracts are most simply interpreted as events initiated in S or G2, and the other two classes are likely to reflect events initiated in G1. Thus, if we count only the simple conversions as illustrated in Figure 1, two-thirds of the spontaneous crossovers have gene conversions indicative of a G1-initiated event, supporting previous conclusions based on a more limited dataset [11]. The numbers of conversion events of all types, as well as their schematic depictions, are given Table S1. About 1/3 of the conversion tracts were more complicated than those predicted by Figure 1. These complex tracts are discussed further in Text S1.

We also examined the conversion tract lengths for the G2- and G1-associated conversions. The median tract lengths of G2- and G1-associated crossovers (Table S1 and S2; Text S1) were 4.7 kb (95% confidence limits of 2.6–9.5 kb) and 14.8 kb (11.7–17.5 kb), respectively. By the Mann-Whitney test, the G1 and G2 conversion tract lengths are significantly different (p<0.0001). The median tract length for all conversion events was 10.6 kb (8.2–13.6 kb).

### Association of conversion tracts with elements of chromosome sequence or structure

From many genetic studies in yeast, it has been shown that the site at which recombination initiates is within or adjacent to the conversion tract [17]. There are a number of elements of chromosome structure in yeast that have been identified as potential chromosome fragile sites or potential hotspots of recombination. We compared our mapping of gene conversion events with the 18 possible recombination-inducing elements listed in Table S4. The locations of some of these elements (G-quadruplexes, replication termination regions, Ty retrotransposons, and long terminal repeats) are depicted in relation to the crossover map in Figure S5.

The methods used for examining potential element enrichments are explained in detail in Text S1. In brief, we determined whether there was a statistically significant excess of elements within the gene conversion tracts relative to that expected based on the known number of elements on the right arm of chromosome IV. In addition to examining all events collectively, we performed data analysis on more exclusive categories: conversion events initiated on the W303a-derived chromosome, conversion events initiated on the YJM789-derived chromosome, G1 conversion events, and G2 conversion events. The statistically significant associations from these analyses are in Table S5.

In the analysis in which all conversions are included, the only significant enrichments were with Ty elements, long terminal repeats (δ elements) that flank Ty elements, and tRNA genes. These same associations were also significant for G1 events and events initiated on the W303a-derived homolog. These associations are expected since the HS3 and HS4 hotspots are G1- and W303a-specific (Figure 4B and 4C). No significant associations were observed between conversion tracts and δ elements *unassociated* with Ty elements. The non-random linkage between Ty elements and tRNA genes in the genome [18] may explain the tRNA enrichment.

Replication forks do not proceed at a constant rate in the genome and replication-pause sites have been associated with a number of chromosome elements including the ribosomal RNA fork barrier, centromeres, tRNA genes, convergent replication forks, G-quadruplex motifs, and highly-transcribed RNA polymerase II genes (reviewed in [20]). We found several similar motifs associated with conversion events. G2 conversion events were significantly associated with replication-termination regions. We also found a significant association between G-quadruplex motifs [21] and G1 conversion events, as well as an association between G-quadruplex motifs and conversion events initiated on the YJM789-derived chromosome; G-quadruplex structures slow replication fork progression [22]. Finally, the conversion events that are initiated on the YJM789-derived chromosome have an over-representation of sites of stalling of the Rrm3p helicase; this helicase is thought to aid replication through genomic regions at which DNA polymerase is paused [20]. We also found some elements of chromosome structure that were negatively associated with the conversion tracts (Table S6).

In addition to looking for associations of various elements *within* the conversion tracts, we looked for associations of these elements with the termini of the tracts. The details of this analysis are described in Text S1. In brief, we examined windows defined by transitions between heterozygous and homozygous markers at the ends of the conversion tracts for over- or under-representation of the chromosome elements described in Table S4. One significant positive association was observed. We found that the LTR elements associated with Ty elements were significantly over-represented at the termini of conversion events. Although it is possible that these regions are preferred sites for the termination of conversion events, there are several alternative interpretations. First, since the YJM789-derived chromosome IV is missing five of the eight Ty elements that are present on the right arm of the W303a-derived chromosome IV [15], several of these LTRs border large regions of heterology. It is possible, therefore, that the conversion tracts are terminated as a consequence of this heterology rather than as a consequence of the sequence/structure of the LTR. Second, HS3 and HS4, the two strongest hotspots, are flanked by Ty-associated LTR events. Thus, these preferred sites for initiating DSBs may lead to preferred sites of tract termination indirectly. A choice among these alteratives will require additional experiments. Finally, it should be emphasized that, despite an enrichment for Ty-associated LTRs at the ends of conversion tracts, the termini of most tracts are not associated with these elements.

## Discussion

In this study, we developed a high-resolution map of spontaneous crossovers on the right arm of chromosome IV, a region that includes about 10% of the yeast genome. Below, we will discuss: 1) the frequency of mitotic crossovers, 2) the distribution of crossover events, 3) the properties of mitotic gene conversion tracts, and 4) the timing of recombination events during the cell cycle.

### Frequency of reciprocal crossovers

The right arm of chromosome IV has about 6.2×10^−5^ crossovers/division or about 6.2×10^−8^ crossovers per kb. As a convenient unit of mitotic crossovers, we suggest that 10^−6^ crossovers/division be defined as one micro Stern (µS), named after Curt Stern, the discoverer of mitotic recombination [23]. We emphasize that this unit is restricted to crossovers that occur between homologs; sister-chromatid exchanges are undetectable by our methods since they do not result in loss of heterozygosity. We calculate that the right arm of chromosome IV has a genetic length of about 62 µS. Extrapolating the data from chromosome IV, we calculate that the yeast genome has a genomic crossover rate of about 6.2×10^−4^/cell division and a genetic map length of about 620 µS. This value is only a rough estimate since the frequency of mitotic recombination hotspots may vary from strain-to-strain, chromosome-to-chromosome, and homolog-to-homolog. However, since mitotic recombination hotspots appear weaker than meiotic recombination hotspots (as described below), some of these variables may have only small effects. For example, although the W303a-derived chromosome IV has more hotspots than the YJM789-derived chromosome IV, the numbers of events initiated on each homolog (determined by which homolog is the donor in the conversion event) are almost identical: 61 events initiated on the W303a-derived homolog and 59 events initiated on the YJM789-derived homolog.

Most previous studies of the rates of spontaneous crossovers utilize a diploid strain that is heterozygous for a *can1* mutation on chromosome V [24]. The heterozygous diploid is sensitive to canavanine, and mitotic crossovers resulting in LOH for the *can1* locus produce cananvanine-resistant derivatives. In several representative studies performed in different genetic backgrounds, the rates of crossovers per division in the 120 kb interval between *CEN5* and *CAN1* were measured as 6×10^−6^ [24], 2×10^−6^ [25], and 2×10^−6^ [11]. These rate estimates are in reasonable agreement with our rate measurement of 6.2×10^−5^ on chromosome IV, since the interval measured in our study is about nine-fold greater than the interval on chromosome V.

Measurements of the rate of mitotic gene conversion events are usually done using diploids that have different non-complementing mutant alleles (heteroalleles) of genes affecting the biosynthesis of an amino acid or nucleotide. The rates of gene conversion, therefore, are estimated by the rate that prototrophic derivatives are produced from the auxotrophic diploid. The rates of heteroallelic gene conversion vary considerably in different studies. Representative rates per division (the heteroallelic gene indicated in parentheses) are: 2.5×10^−7^ (*arg4*) [26], 2.6×10^−6^ (*trp5*) [27] and 3×10^−7^ (*leu2*) [28]. Since gene conversion events are initiated by DSBs, the frequency of these events will be affected by the hotspot distribution. In addition, the distance between the mutant substitutions would be expected to influence the rate of gene conversion with this assay. It is important to note that gene conversion requires a recombinogenic DSB near the heteroalleles, whereas the crossover assays detect DSBs distributed throughout the region between the reporter gene and the centromere. In the present study, our analysis of conversion events was restricted to those associated with crossovers on chromosome IV. In a previous study, using microarrays that covered the entire yeast genome, no unselected spontaneous gene conversion events were detected in thirteen strains examined [16].

The yeast genome has a genetic length of about 4100 cM (SGD), representing about 81 meiotic crossovers/division. Thus, the rate of crossovers in meiosis is about 10^5^-fold higher than the mitotic rate. Although the rate of mitotic crossovers/division is low relative to the meiotic rate, since the number of mitotic divisions is likely to greatly exceed the number of meiotic divisions, mitotic crossovers are likely to be a potent mechanism for generating novel combinations of alleles.

### General considerations concerning the distribution of crossovers

The distributions of mitotic crossovers and their associated gene conversion tracts are shown in Figure 3 and Figure 4. Two points concerning the distribution should be emphasized. First, by our statistical analysis, not all of the peaks labeled as hotspots have significantly elevated levels of recombination. Second, for conversion-associated crossovers, the position of the initiating DNA lesion can be anywhere within the conversion tract. Previously, we showed that 230 repeats of the GAA trinucleotide was a hotspot for DSB formation [29]. In individual conversion events associated with this hotspot, the conversion tracts were propagated either toward the centromere, away from the centromere or bidirectionally from the tract. Our mapping of DNA lesions and the resolution of the mitotic recombination map, therefore, are limited by the size of the conversion tracts rather than the distribution of SNPs.

An important conclusion from our analysis is that two homologs can differ significantly in their distribution of recombination events (Figure 4B and Figure 5). From the data shown in Figure 4B, it appears that HS1, HS3, and HS4 are hotspots on the W303a-derived chromosome, HS2 and HS6 are hotspots on the YJM789-derived chromosomes, and HS5 and HS7 are hotspots on both homologs. Thus, construction of a universal genetic map of mitotic events is difficult.

Meiotic recombination events have been mapped throughout the genome using various types of microarrays [30]–[35]. The patterns of meiotic and mitotic recombination events on the right arm of chromosome IV show no evident similarity (Figure S6). Since meiotic recombination are initiated by meiosis-specific cleavage of the genome by Spo11p and the DNA lesions that initiate mitotic recombination are likely to have a variety of sources (discussed further below), this difference in meiotic and mitotic patterns of recombination is expected. The levels of meiosis-specific DSBs at different places in the genome vary at least 400-fold [34]. We can estimate the relative “heat” of a chromosome region by determining the number of times that SNPs within that region are included within conversion tracts. For example, the peak SNP in HS4 (the strongest hotspot) was included in eleven recombination events (Figure 4). Since the average number of events for all SNPs throughout the right arm of chromosome IV is two, HS4 is only about five times more active than an average region. Thus, our data suggest that hotspots and coldspots are more pronounced for meiotic than for mitotic recombination.

### Inverted repeats as hotspots for mitotic recombination

Both HS3 and HS4 co-localize with inverted Ty elements. Inverted repeats have been previously shown to be hotspots for certain types of mitotic recombination [5], [36]. Most of these studies have involved non-yeast DNA sequences inserted into the genome by transformation. In our previous study [7], a hotspot for chromosome rearrangements on chromosome III in cells that have low levels of DNA polymerase alpha was an inverted pair of Ty elements separated by about 280 bp. In contrast, HS3 and HS4 function as hotspots in cells with wild-type levels of DNA polymerases.

The conversion tracts associated with inverted repeats at HS3 and HS4 are primarily 4∶0 or hybrid events, arguing that the recombinogenic lesion is formed prior to replication. Two types of secondary structures have been associated with inverted repeats: cruciforms and hairpins. Evidence for cruciform processing (presumably by a Holliday junction-like resolvase) resulting in two hairpin-capped ends has been obtained [5]. However, spacers greater than 20 bases substantially inhibit cruciform formation in *E. coli* [37]–[39], and reduce the recombinogenic properties of palindromes in yeast [40]. An alternative model is that a single-stranded nick near the junction of the inverted repeats is processed into a single-stranded DNA gap, allowing formation of a hairpin on the intact DNA strand. The loop formed by the spacer in the hairpin could then be nicked by a nuclease such as the Mre11p complex [5]. We do not know the source of the nick that initiates hairpin formation. It is possible that DNA supercoils produced by Ty transcription are nicked by topoisomerases [41]. Our deletion analysis, however, demonstrates that convergent transcription of *both* Ty elements is not necessary for HS4 activity. It is also possible that the nick that initiates hairpin formation could represent a lesion generated by mismatch repair. Since the hotspot activity of HS3 and HS4 appears G1-specific, it is likely that the initiating nick occurs in G1.

### Mitotic recombination events that are initiated independently of inverted repeats

In addition to inverted repeats, several other factors have been associated with mitotic recombination or DSB formation. Using several different assays, researchers have found that elevated levels of transcription stimulate recombination (reviewed in [1]). Since mutations in many enzymes involved in DNA replication result in a hyper-Rec phenotype, it is likely that some spontaneous recombination events result from DNA lesions generated during DNA replication [42]. It has been shown that some DNA sequences or motifs are associated with slow or stalled replication forks [42]. Consequently, we analyzed our conversion tracts to determine if there was an over-representation of these structure/sequence motifs. We found that G4 quadruplex motifs were significantly associated with G1-initiated DSBs (Table S5). In yeast cells, G4 motifs have been shown previously to be enriched in areas of the genome that have high γ-H2AX binding, a signal associated with DNA damage [21]. In our study, the events initiated during S or G2 of the cell cycle were significantly associated with replication-termination (TER) regions. This association is especially intriguing because sister chromatids are intercalated following replication, and need to be resolved by Top2p [43], [44]. Since Top2p induces DSBs to allow decatenation of sister-chromatids, it is possible that some of these Top2p induced cleavages are misrepaired, leading to homologous recombination.

### Conversion tract lengths

The median conversion tract length for all analyzed crossovers on the right arm of chromosome IV was 10.6 kb (95% confidence limits: 8.2–13.6 kb). As discussed in Results, we found that conversion tract lengths associated with G1-initiated breaks were significantly longer than G2-initiated breaks. Since the crossovers of both G1- and G2-initiated events occur in G2, the broken ends generated by a G1-initiated lesion have a longer time for 5′-to-3′ resection prior to repair of the DSBs. In addition, the G1-initiated events involve the repair of two broken chromatids. If the conversion tracts associated with these two repair events are propagated in different directions, we would expect that the G1-initiated conversions would be longer than those initiated in G2. As noted previously [11], the mitotic conversion tracts characterized in our system are considerably longer than the average meiotic conversion tract of about 2 kb [45].

One of the unique characteristics of recombination events associated with HS4 is that their conversion tracts are substantially longer than those that occur at other locations along chromosome IV. The median conversion tract length associated with HS4 is 48.4 kb (95% confidence limits: 17.1–118.3 kb), much longer than the median tract length for all conversions (10.6 kb) and longer than the median length of G1-induced conversions (14 kb). One factor that would be expected to affect tract length is that HS3 has two intact Ty elements on the W303a-derived homolog whereas the YJM789-derived homolog has only a 2 kb fragment of a Ty (Figure 6, Figure S3). Thus, a DSB formed in this hemizygous insertion on the W303a-derived chromosome would need to be processed about 10 kb before exposing homology on the YJM789-derived chromosome. In addition, since the homology would be located internally on the resected strand, a recombination intermediate with single-stranded branches would be formed [17]. Removal of these single-stranded branches likely requires the nucleotide excision repair proteins Rad1p and Rad10p [46]–[47], as well as the mismatch repair proteins Msh2p and Msh3p [48]–[51]. Thus, the hotspot activity of HS4 would also be dependent on these proteins. It is possible that the more extensive processing of the DSBs occurring in HS4 delays completion of the crossover, resulting in a longer crossover-associated gene conversion tract.

### DSBs initiated during G1 versus G2 of the cell cycle

As described above, about two-thirds of the observed conversion events involve the repair of two sister chromatids broken at approximately the same position. We infer these events are a consequence of a G1-induced DSB on one of the homologs, followed by replication of the broken chromosome. The repair of two broken chromatids subsequently occurs in G2 (Figure 1B and 1C). This model is supported by the observation that γ-irradiation of G1-synchronized yeast cells results in 4∶0 and 4∶0/3∶1 hybrid tracts, whereas irradiation of G2-synchronized cells results in primarily 3∶1 tracts [12]. Although we prefer the interpretation that these events are initiated by a G1 DSB, we cannot rule out the possibility that a DNA lesion produces two broken chromatids during DNA synthesis. Conversion tracts more complicated than those depicted in Figure 1 were observed. These are discussed in Text S1, and a mechanism to explain a class E2 (Table S1) conversion tract is presented in Figure S7.

**Figure 7:**
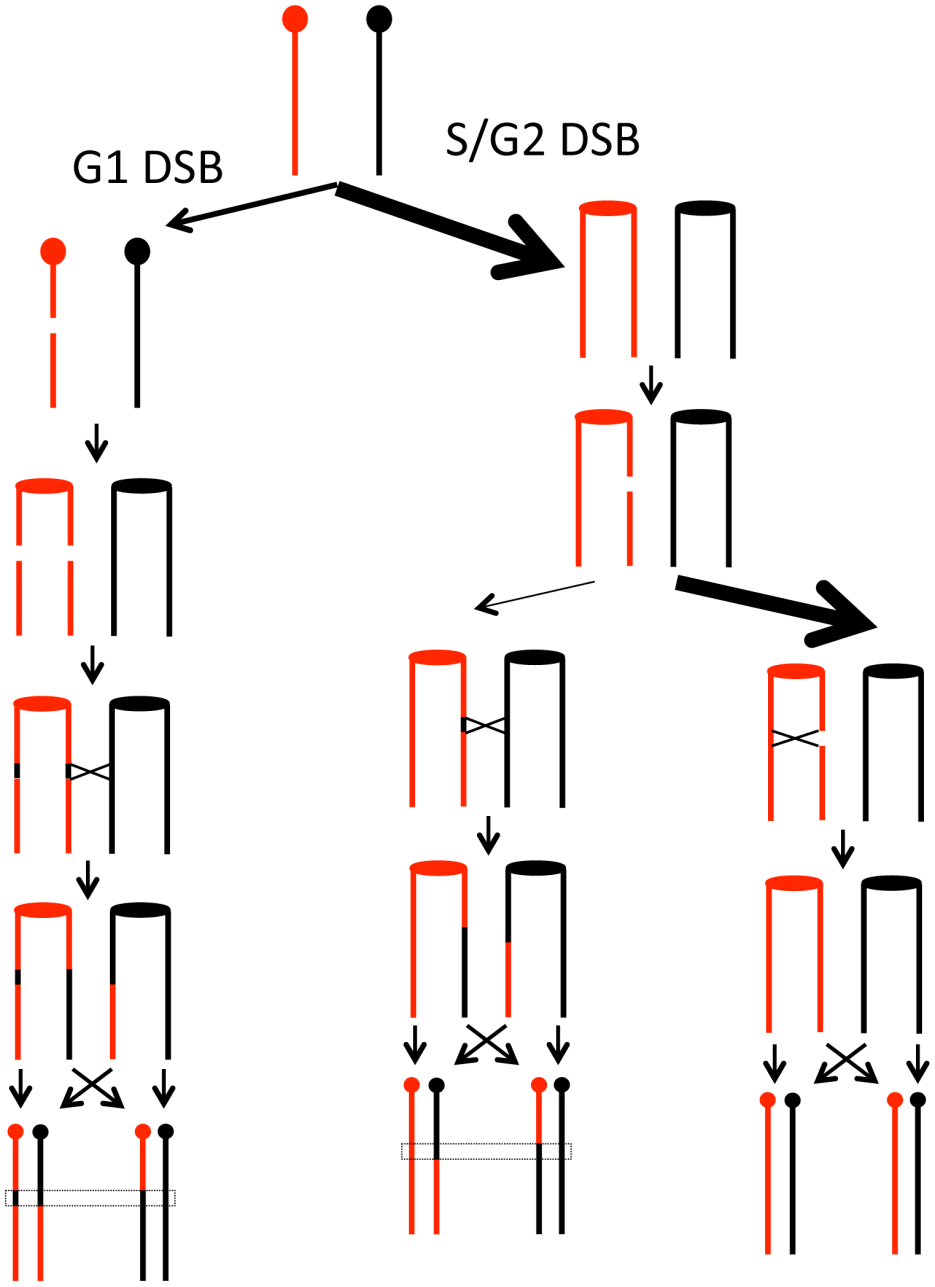
Model for homologous recombination repair choices depending on the timing of the initiating DSB lesion. We suggest that most spontaneous DSBs occur during S- or G2 of the cell cycle as indicated by the relative widths of the arrows. The DSBs that occur in G1, however, are repaired preferentially by recombination with the homolog, whereas the DSBs that occur in G2 are repaired preferentially by recombination with the sister chromatids. The rationale for these preferences is given in the text.

Although about two-thirds of the LOH-inducing events were caused by G1-DSBs in our study, it is likely that more spontaneous DSBs occur in S than in G1. Rad52 foci, indicative of HR, are more frequent in the S- and G2-phases than in G1 [52]. A related observation is that the extent of resection of broken ends is substantially greater in G2 than in G1 [53], [54]. One simple model that reconciles these observations is shown in Figure 7. We suggest that most spontaneous DSBs are generated in S as a consequence of broken replication forks. These DSBs are preferentially repaired by sister chromatid recombination with only a small fraction involving an interaction with the homolog; this preference is likely to be ensured by sister-chromatid cohesion [55]. In yeast, DSBs generated by ionizing radiation during G2 of the cell cycle are usually repaired using the sister chromatid as the template [56]; such events are undetectable by our analysis. In contrast, the DSBs generated in G1, even though less frequent than DSBs initiated in S, are preferentially repaired by recombination between the homologs, because both sister chromatids have a break at the same location. Thus, the model accounts for the preferential the use of the homolog in a G1-initiated event.

In summary, our analysis shows that mitotic recombination events are distributed broadly along the chromosome. One well-defined class of hotspots is a consequence of secondary structure formation between inverted repeats. We suggest that most of the recombination events leading to loss of heterozygosity are initiated by a DSB in G1 since DSBs that occur in S or G2 are usually repaired by sister-chromatid exchanges.

## Materials and Methods

### Strains and construction

A list of all strains used in this study is in Table S7, and a list of all primers used is in Table S8, unless otherwise specified. Mapping of crossovers was performed with the strain JSC25 (*MATa/MATα::HYG leu2-3,112/LEU2 his3-11,15/HIS3 ura3-1/ura3 GAL2/gal2 ade2-1/ade2-1 trp1-1/TRP can1-100::NAT/CAN1::NAT RAD5/RAD5 IV1510386::KANMX-can1-100/IVI1510386::SUP4-o*), a diploid generated by mating the haploids W303a and YJM789. Details of strain constructions are in Text S1.

### Media

All media were prepared using standard recipes [57], except that the SD-Arg plates contained only 10 ìg/mL of adenine, as previously described [14].

### Assay for crossovers

To detect crossovers, we struck JSC25 to generate single colonies on rich solid medium (YPD plates). The cells were grown for three days at 30°. A single colony was then resuspended in water and plated onto SD-Arg plates at a density of about 3000 cells per plate. Plates were incubated at 25° for three days and then at 4° for an additional day to let the red pigmentation develop. Red/white sectors were detected using a dissecting microscope. In colonies in which the red sector was at least one-eighth of the colony, we purified colonies from both the red and white sides of the sector. We confirmed that the red colonies were Ade^−^ (due to loss of *SUP4-o*) and that the white colonies were Gen^S^ (due to loss of the *KANMX* gene). Single red and single white colonies from each sectored colony were subsequently analyzed.

### Microarray methods and analysis

Microarray samples were prepared, hybridized, and analyzed as previously described [16]. Briefly, agarose plugs containing genomic DNA were prepared from strains derived from the sectored colonies and from the control diploid reference strain. DNA from both the experimental and reference strains was sonicated to yield DNA fragments of about 200–400 bp; these samples were labeled with Cy5-dUTP and Cy3-dUTP, respectively. DNA samples from the experimental and control strains were mixed and competitively hybridized to SNP microarrays. Following washing and scanning of the arrays, probe signals were analyzed as ratios of hybridization of the experimental and control samples for each SNP-specific oligonucleotide represented on the arrays.

### Microarray design

The chromosome IV SNP microarray was designed and optimized following the principles described previously [16]. For the majority of the probes, the SNP is located in the center of the oligonucleotide, and the melting temperature for the oligonucleotide/genomic DNA hybrid is between 55 and 59°C. Minor deviations from these principles are described in Text S1. The location and sequence of each SNP on the microarray are listed in Table S9.

### Assay of recombination activity of HS4 containing inverted repeats

Recombination between the *URA3* and *HYG* markers was assayed by monitoring the *HYG* gene in 5-FOA^R^ strains. Each strain was struck for single colonies on YPD plates. After incubating the cultures at 30° for 30 hours, we made patches of individual colonies on plates containing medium with 5-FOA. The plates were then incubated for two days at 30°. A single 5-FOA^R^ colony from each patch was then transferred as a patch to YPD plates. After one day of growth at 30°, the samples were replica-plated to YPD plates containing hygromycin. About 400 5-FOA^R^ colonies were analyzed for each strain.

### Statistical analysis

We used chi-square goodness-of-fit tests to compare expected values with observed values. VassarStats (http://vassarstats.net/) and Microsoft Excel were used for this statistical test. We used Table B11 of [58] to calculate 95% confidence intervals on median estimates of conversion tract length. The Mann-Whitney test from the VassarStats website was used to compare conversion tract lengths. Data were corrected for multiple comparisons in the element enrichment analysis [59].

## Supporting Information

Figure S1 Mapping of a crossover with an associated 3∶1/4∶0/3∶1 conversion event by SNP microarrays. The depiction of this event is the same as in Figure 2. A. Low-resolution depiction of the crossover. In both the red (top plot) and white (bottom plot) sectors, there is a transition between heterozygosity and homozygosity at approximately SGD coordinate 700 kb, although it is evident in JSC125 SP 112W that there are at least two transitions. B. High-resolution depiction of 3∶1/4∶0/3∶0 conversion associated with a reciprocal crossover. A comparison between the patterns of SNP heterozygosity and homozygosity demonstrate that the crossover in this sectored colony was associated with a 3∶1/4∶0/3∶1 conversion tract. The 3∶1 segments of the tract are included within the green rectangles and the 4∶0 portion is outlined in the red triangles.
(TIF)

Figure S2 Distribution of JSC25 crossovers into six intervals along the right arm of chromosome IV. As described in the text, we grouped the observed crossovers into six bins along the right arm of chromosome IV. Five of these bins were 200 kb, and the last bin was 75 kb. The Y-axis shows the number of events/kb/bin. The X-axis shows the SGD coordinates of each bin. A. For this analysis, all crossovers were examined without regard to which homolog had the initiating DNA lesion or whether the conversion event was initiated by a G1- or G2/S-associated DSB. B. For this analysis, we classified the crossovers in each bin into four groups: crossovers initiated on the W303a-derived chromosome in G1 (red), crossovers initiated on the W303a-derived chromosome in G2 (pink), crossovers initiated on the YJM789-derived chromosome in G1 (black), and crossovers initiated on the YJM789-derived chromosome in G2 (gray).
(TIF)

Figure S3 The inverted pair of Ty elements at SGD coordinate 980 kb are present on the W303a-, but not the YJM789-derived homolog [14]. A. Top: Structure of the Ty inverted repeat located near 980 kb on the W303a-derived chromosome IV. F_1_, F_2_, R_1_, and R_2_ are primers used to diagnose the existence of these repeats on the W303a and YJM789 homologs. Primers F_1_, R_1_, F_2_, and R_2_ are listed in Table S8 as IV 980403 F, Ty2 R, Ty1.2 R, and IV 993256 R, respectively. Bottom: Gel analysis of the PCR products using F_1_/R_1_ and F_2_/R_2_ primer pairs and the indicated template DNA; S288c is very similar in DNA sequence to W303a [8]. B. Gel analysis of the PCR reaction using primer pair F_1_/R_2_. This analysis indicates the existence of a partial Ty element in the YJM789 genome. C. Summary of the structure of the inverted Ty repeats at HS4 in the W303a-derived homolog and the absence of this structure in the YJM789-derived homolog. These results are also consistent with the genomic sequencing of the two strains.
(TIF)

Figure S4 Potential “hairpin” DNA structures that could be formed by inverted Ty elements at HS4. Bases with homology to Ty or delta sequences are shown in red; bases with no homology to Ty are shown in black. A. Sequence of 98 bases from one strand of the DNA at the center of the inverted Ty pair (SGD coordinates 987091–987188) at HS4. Regions of possible intrastrand pairing are indicated by brackets. B. Secondary structure formed by HS4 with a terminal 25 bp spacer and an unpaired 9 base loop. C. Secondary structure formed by HS4 with a 66 bp spacer.
(TIF)

Figure S5 Location of possible recombination-inducing elements relative to chromosome IV recombination events. A. Recombination profile of the right arm of chromosome IV. These are the same data that are presented in Figure 4A. B. The locations of G-quadruplex motifs [17], Ty retrotransposons (obtained from SGD), long terminal repeats (obtained from SGD), replication termination regions [18], and palindromes [15] on the right arm of chromosome IV are indicated by blue, red, green, purple, and black colored lines, respectively.
(TIF)

Figure S6 Comparison of mitotic and meiotic recombination maps on the right arm of chromosome IV. The Y-axis in the figure shows the number of times individual SNPs are included in a crossover-associated gene conversion. The Y-axis shows SGD coordinates on chromosome IV. A. Summary of our mapping of mitotic events in JSC25. B. Summary of the mapping of meiotic events in a closely-related diploid strain by [14].
(TIF)

Figure S7 Generation of a complex conversion tract by “patchy” repair of mismatches in a heteroduplexes. This figure shows one mechanism for generating the complex gene conversion tract shown as the Class E2 event of Table S1. The homologs are depicted as double-stranded DNA molecules with the W303a-derived homolog shown as red lines and the YJM789-derived homolog shown as black lines. Recombination is initiated by a DSB on chromatid 2 in G2. The broken chromatid undergoes 5′ to 3′ resection (step 1), followed by strand invasion of the left end into chromatid 3 (step 2). DNA synthesis initiated by the broken end (shown as dotted lines), displaces one of the strands of chromatid 3, allowing second-end capture and DNA synthesis by the right hand broken end. The resulting double Holliday junction has two regions of heteroduplex (shown in blue rectangles), and is processed by cleaving the left and right junctions (cleavage sites shown by triangles) (step 3). Following junction resolution, the chromosome regions flanking the heteroduplexes are in the recombined configuration (step 4). In step 5, the mismatches within the two heteroduplexes are repaired. All of the mismatches in the heteroduplex on chromatid 3 are repaired in the same direction (duplicating YJM789-derived SNPs), whereas the heteroduplex on chromatid 4 undergoes “patchy” repair. Segregation of chromatids 1 and 3 into one cell and chromatids 2 and 4 into the other result in a crossover associated with the complex conversion tract depicted as Class E2 in Table S1.
(TIF)

Table S1 Depictions of conversion tracts associated with reciprocal crossovers. All of the different types of conversion tracts observed in this study are shown in this table. For each conversion tract, the top colored line depicts the red side of the sector and the bottom line depicts the white side of the sector. Green, red, and black indicate heterozygosity for SNPs, homozygosity for W303a-derived SNPs, and homozygosity for YJM789-derived SNPs, respectively. Lowercase letters located above and below the conversion tract mark transitions within the conversion tract. The coordinates of the SNPs on either side of the transitions are listed in Table S2. The blue boxes enclose the regions that are likely to be associated with DSB formation as described in Text S1. Our interpretations of subclasses as representing G1- or G2-initiated events are indicated by asterisks to the right of the subclass names. One asterisk and two asterisks indicate a G1- and G2-initiated DSB, respectively. The lengths of the conversion tracts are not drawn to scale.
(XLSX)

Table S2 SGD coordinates for crossover and conversion transitions on chromosome IV. ^1^The classes of events are defined in Table S1. ^2^The lower case letters refer to transitions between heterozygous and homozygous regions as shown in Table S1. ^3^These numbers represent SGD coordinates of SNPs located on each side of the transition. It should be noted that these coordinates are based on SGD coordinates from Feb. 2010, and some of these coordinates may be different from those currently displayed in SGD.
(DOCX)

Table S3 Coordinates used to represent gene conversion tracts as single points. For some of the analysis of hotspots, the conversion tracts were represented as to single points. The method for calculating the single point is described in Text S1. The description of each sectored colony is in Tables S1 and S2.
(XLSX)

Table S4 Chromosome elements examined for their representation in mitotic gene conversion tracts. ^1^For most of the listed elements, we examined the number of such elements between *CEN4* and the markers near the right telomere used to diagnose crossovers. ^2^Palindromes that were at least 16 bp are counted. ^3^The repeats examined were in the size range of 2 to 213 bp, with a minimum repeat tract of 24 bp. ^4^This database is accessible at the Website: https://tandem.bu.edu/cgi-bin/trdb/trdb.exe ^5^The minimal number of repeats/tract was 8. ^6^The G4 motifs used in this analysis were four tracts of 3 G's separated by spacers of less than 25 bp. ^7^Transcription levels were measured for 451 of the 565 ORFs on the right arm of chromosome IV in [19]. Most of these genes were single-copy sequences, since measuring transcription from repeated genes with very similar sequences is difficult. We identified the twenty single-copy genes in this region with the highest level of transcription. ^8^Peaks of accumulation of gamma-H2AX within the genome were mapped; such regions are often associated with DNA damage [20]. ^9^Rrm3p is a helicase involved in promoting replication through replication-pause sites [21]. ^10^We identified the locations of the 17 longest intergenic regions of a total of 554 on the right arm of chromosome IV. ^11^ARS elements were identified in which the genes flanking the ARS have transcripts that converge on the ARS.
(DOCX)

Table S5 Chromosome elements that are over-represented in crossover-associated gene conversion tracts in JSC25. ^1^As described in the text, we used four related methods of analysis to determine whether various structure/sequence chromosome motifs (listed in Table S4) were over-represented in gene conversion tracts associated with crossovers on chromosome IV. ^2^The data examined by Methods 1-4 were: JSC25 all (G1 and G2 events initiated on either W303a- and YJM789-derived homologs), JSC25 G1 (G1 events initiated on either W303a- and YJM789-derived homologs), JSC25 G2 (G2 events initiated on either W303a- and YJM789-derived homologs), W303a all (G1 and G2 events initiated on the W303-derived homologs), and YJM789 all (G1 and G2 events initiated on the YJM789-derived homologs). ^3^Rrm3p is a helicase that promotes replication fork progression through regions at which the forks are paused [21].
(DOCX)

Table S6 Chromosome elements that are under-represented in crossover-associated gene conversion tracts in JSC25. ^1^As described in the text, we used four related methods of analysis to determine whether various structure/sequence chromosome motifs (listed in Table S4) were under-represented in gene conversion tracts associated with crossovers on chromosome IV. ^2^The data examined by Methods 1–4 were: JSC25 all (G1 and G2 events initiated on either W303a- and YJM789-derived homologs), JSC25 G1 (G1 events initiated on either W303a- and YJM789-derived homologs), JSC25 G2 (G2 events initiated on either W303a- and YJM789-derived homologs), W303a all (G1 and G2 events initiated on the W303-derived homologs), and YJM789 all (G1 and G2 events initiated on the YJM789-derived homologs).
(DOCX)

Table S7 Strain list and strain constructions. ^1^ Standard gene nomenclature is used with a few exceptions. An insertion unaccompanied by a deletion is indicated by a double colon; a replacement is indicated by a Δ, followed by a double colon and the name of the replacing gene. If an insertion is placed within a genomic sequence unassociated with a gene name, we indicate the position of the centromere-proximal base followed by a double colon and the name of the inserted gene. For example, *IV10132176::URA3* indicates that the *URA3* gene was inserted between bases 10132176 and 10132177 on chromosome IV. For diploid genotypes, the genotype of the W303a-derived haploid is shown above the diagonal and the genotype of the YJM789-derived haploid is shown below the diagonal. The HS4 hotspot (inverted Ty elements) is missing on the YJM789-derived homology; the nomenclature used to describe the missing Ty elements in the genotypes above is: *ydrwTy2-3Δ*.
(DOCX)

Table S8 Names and sequences of primers used in strain constructions and genome analysis. ^1^Primer sequences are written 5′ to 3′.
(DOCX)

Table S9 Sequences and coordinates of probes for the chromosome IV SNP microarray. This table describes the probes that compose the chromosome IV SNP microarray. The first column designates the names of each probe on the array. The second column gives the sequence for each probe on the array, and the third column designates which background the probe is specific for. Each probe is part of a group of four probes, one specific for each strand of each strain background. The fourth and fifth columns give the coordinates of the 5′ and 3′ ends of each probe group as determined by the Watson strand, respectively. Coordinate numbers are based on the January 2010 SGD sequences.
(XLSX)

Text S1 Supplemental materials and methods and discussions.
(DOCX)
