# Supplementary figures and images for: High-Resolution Mapping of Spontaneous Mitotic Recombination Hotspots on the 1.1 Mb Arm of Yeast Chromosome IV
Source: PLoS Genet. 2013 Apr 4;9(4):e1003434. doi: 10.1371/journal.pgen.1003434 (PMC3616911; doi:10.1371/journal.pgen.1003434)

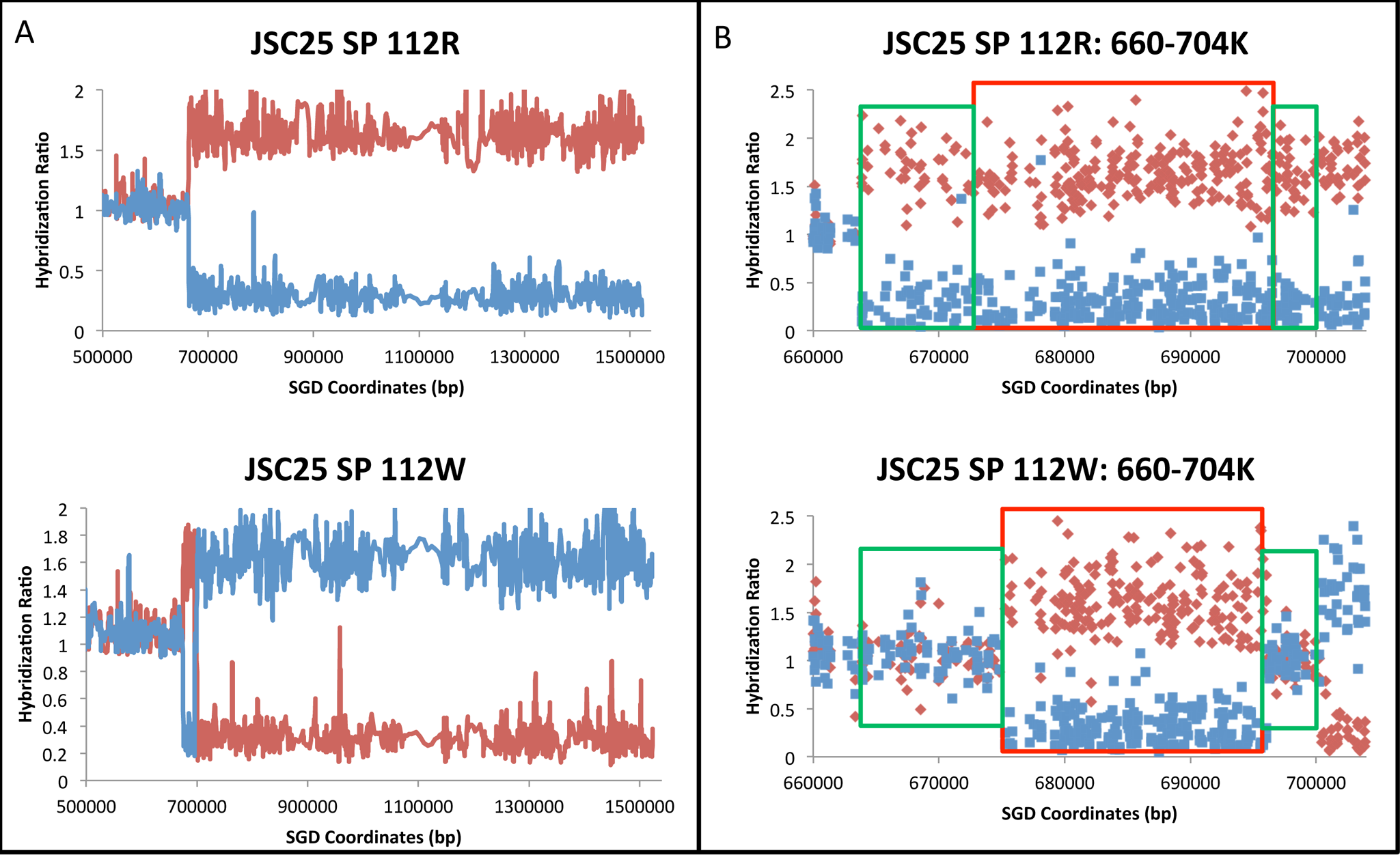

Supplement: Figure S1 — Mapping of a crossover with an associated 3∶1/4∶0/3∶1 conversion event by SNP microarrays. The depiction of this event is the same as in Figure 2. A. Low-resolution depiction of the crossover. In both the red (top plot) and white (bottom plot) sectors, there is a transition between heterozygosity and homozygosity at approximately SGD coordinate 700 kb, although it is evident in JSC125 SP 112W that there are at least two transitions. B. High-resolution depiction of 3∶1/4∶0/3∶0 conversion associated with a reciprocal crossover. A comparison between the patterns of SNP heterozygosity and homozygosity demonstrate that the crossover in this sectored colony was associated with a 3∶1/4∶0/3∶1 conversion tract. The 3∶1 segments of the tract are included within the green rectangles and the 4∶0 portion is outlined in the red triangles. (TIF) [file pgen.1003434.s001.tif]

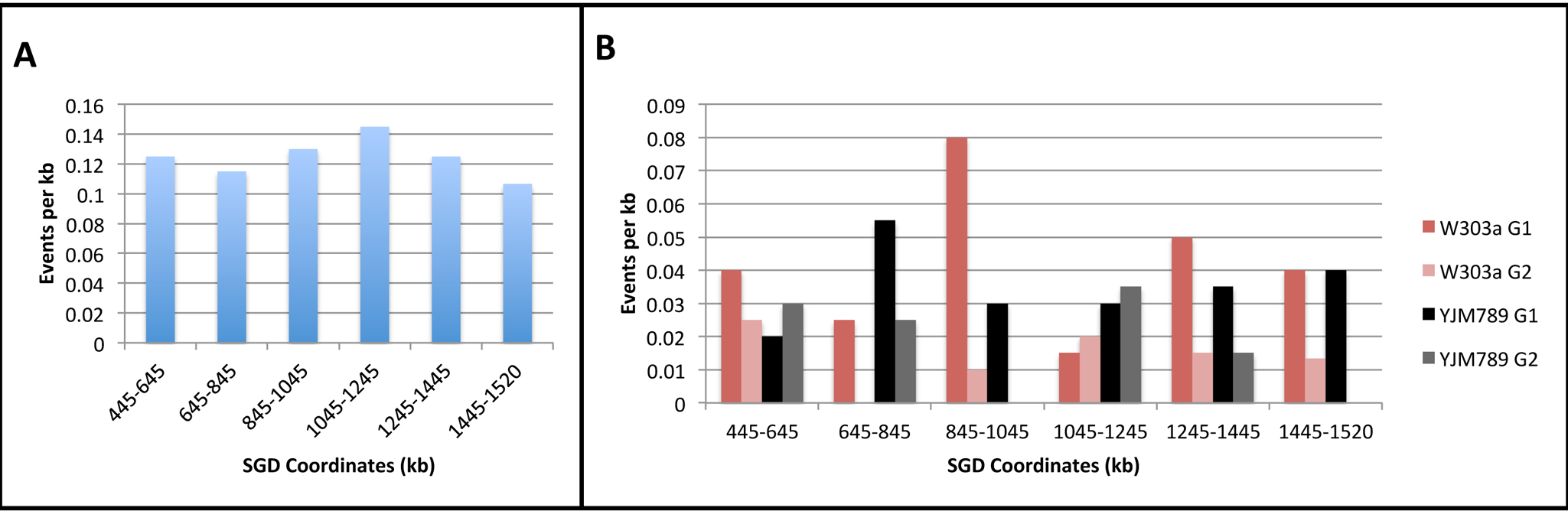

Supplement: Figure S2 — Distribution of JSC25 crossovers into six intervals along the right arm of chromosome IV. As described in the text, we grouped the observed crossovers into six bins along the right arm of chromosome IV. Five of these bins were 200 kb, and the last bin was 75 kb. The Y-axis shows the number of events/kb/bin. The X-axis shows the SGD coordinates of each bin. A. For this analysis, all crossovers were examined without regard to which homolog had the initiating DNA lesion or whether the conversion event was initiated by a G1- or G2/S-associated DSB. B. For this analysis, we classified the crossovers in each bin into four groups: crossovers initiated on the W303a-derived chromosome in G1 (red), crossovers initiated on the W303a-derived chromosome in G2 (pink), crossovers initiated on the YJM789-derived chromosome in G1 (black), and crossovers initiated on the YJM789-derived chromosome in G2 (gray). (TIF) [file pgen.1003434.s002.tif]

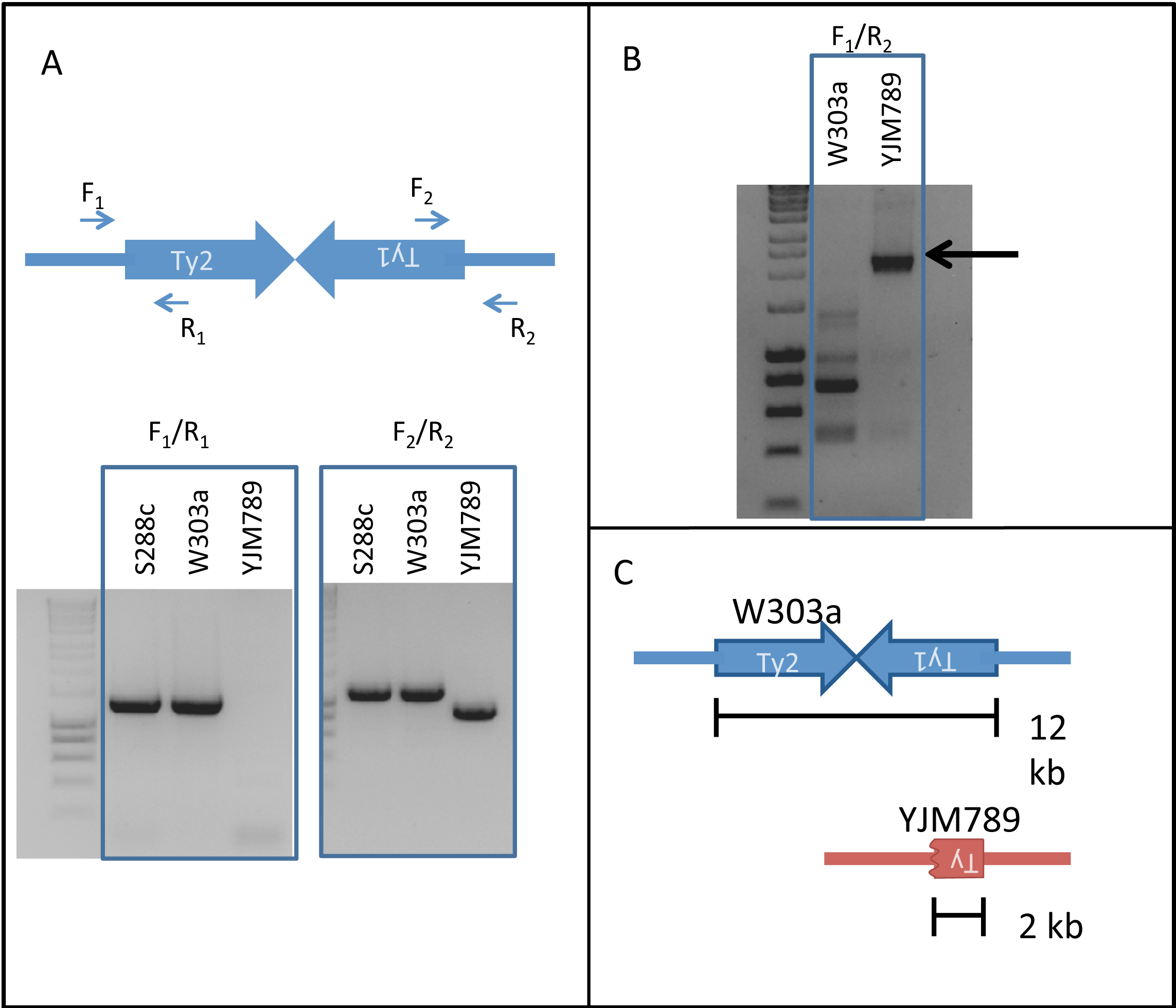

Supplement: Figure S3 — The inverted pair of Ty elements at SGD coordinate 980 kb are present on the W303a-, but not the YJM789-derived homolog [14]. A. Top: Structure of the Ty inverted repeat located near 980 kb on the W303a-derived chromosome IV. F1, F2, R1, and R2 are primers used to diagnose the existence of these repeats on the W303a and YJM789 homologs. Primers F1, R1, F2, and R2 are listed in Table S8 as IV 980403 F, Ty2 R, Ty1.2 R, and IV 993256 R, respectively. Bottom: Gel analysis of the PCR products using F1/R1 and F2/R2 primer pairs and the indicated template DNA; S288c is very similar in DNA sequence to W303a [8]. B. Gel analysis of the PCR reaction using primer pair F1/R2. This analysis indicates the existence of a partial Ty element in the YJM789 genome. C. Summary of the structure of the inverted Ty repeats at HS4 in the W303a-derived homolog and the absence of this structure in the YJM789-derived homolog. These results are also consistent with the genomic sequencing of the two strains. (TIF) [file pgen.1003434.s003.tif]

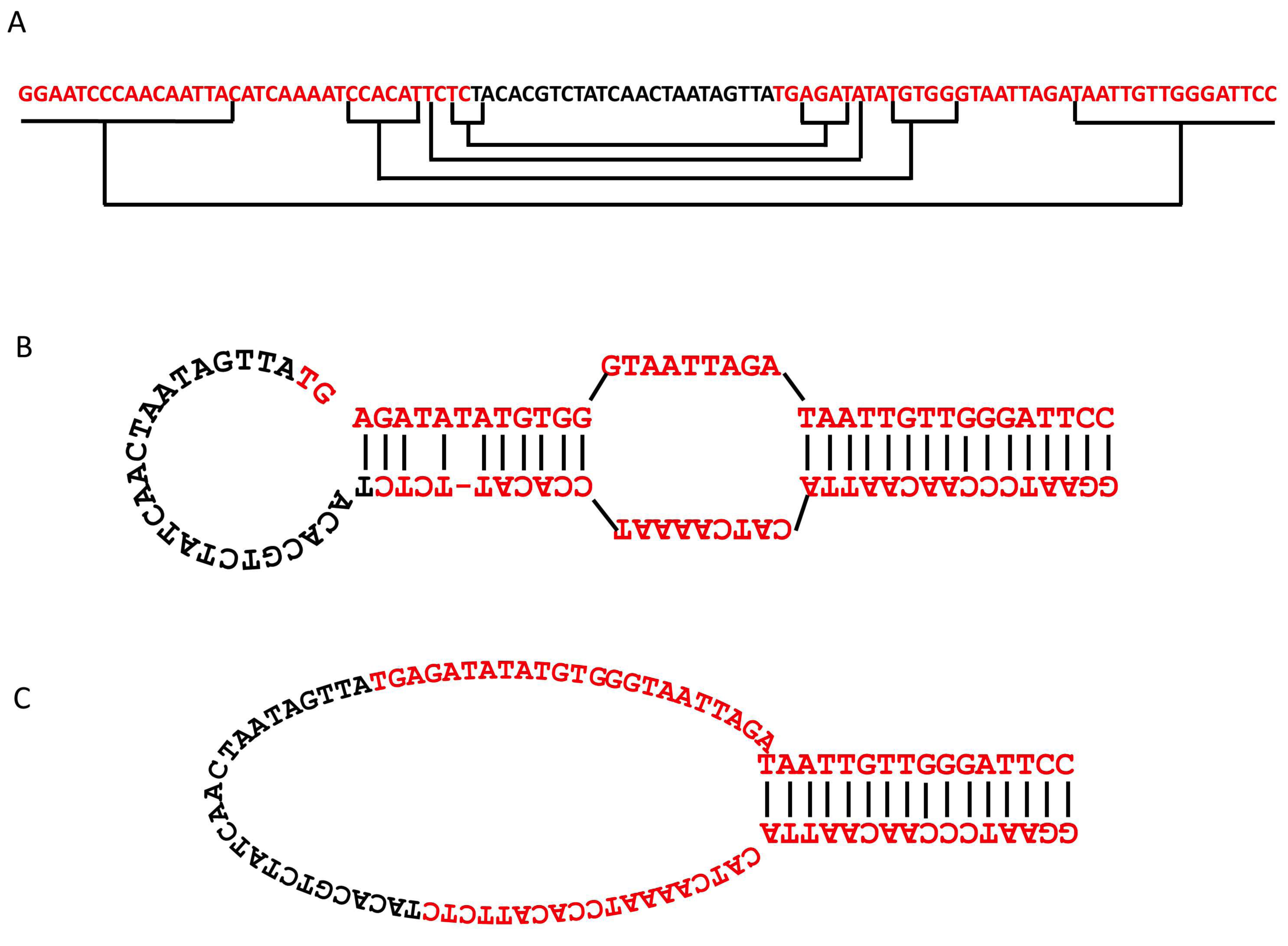

Supplement: Figure S4 — Potential “hairpin” DNA structures that could be formed by inverted Ty elements at HS4. Bases with homology to Ty or delta sequences are shown in red; bases with no homology to Ty are shown in black. A. Sequence of 98 bases from one strand of the DNA at the center of the inverted Ty pair (SGD coordinates 987091–987188) at HS4. Regions of possible intrastrand pairing are indicated by brackets. B. Secondary structure formed by HS4 with a terminal 25 bp spacer and an unpaired 9 base loop. C. Secondary structure formed by HS4 with a 66 bp spacer. (TIF) [file pgen.1003434.s004.tif]

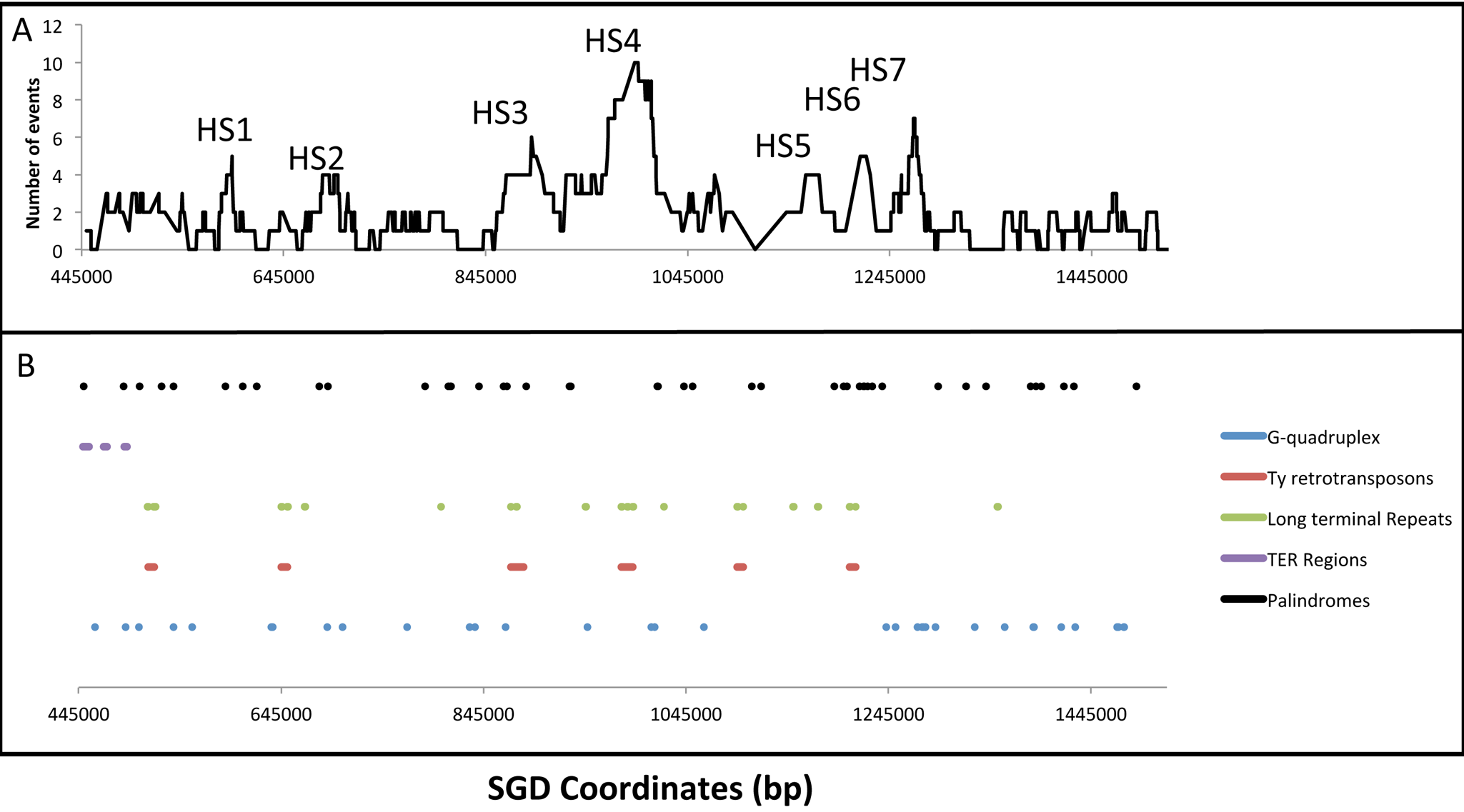

Supplement: Figure S5 — Location of possible recombination-inducing elements relative to chromosome IV recombination events. A. Recombination profile of the right arm of chromosome IV. These are the same data that are presented in Figure 4A. B. The locations of G-quadruplex motifs [17], Ty retrotransposons (obtained from SGD), long terminal repeats (obtained from SGD), replication termination regions [18], and palindromes [15] on the right arm of chromosome IV are indicated by blue, red, green, purple, and black colored lines, respectively. (TIF) [file pgen.1003434.s005.tif]

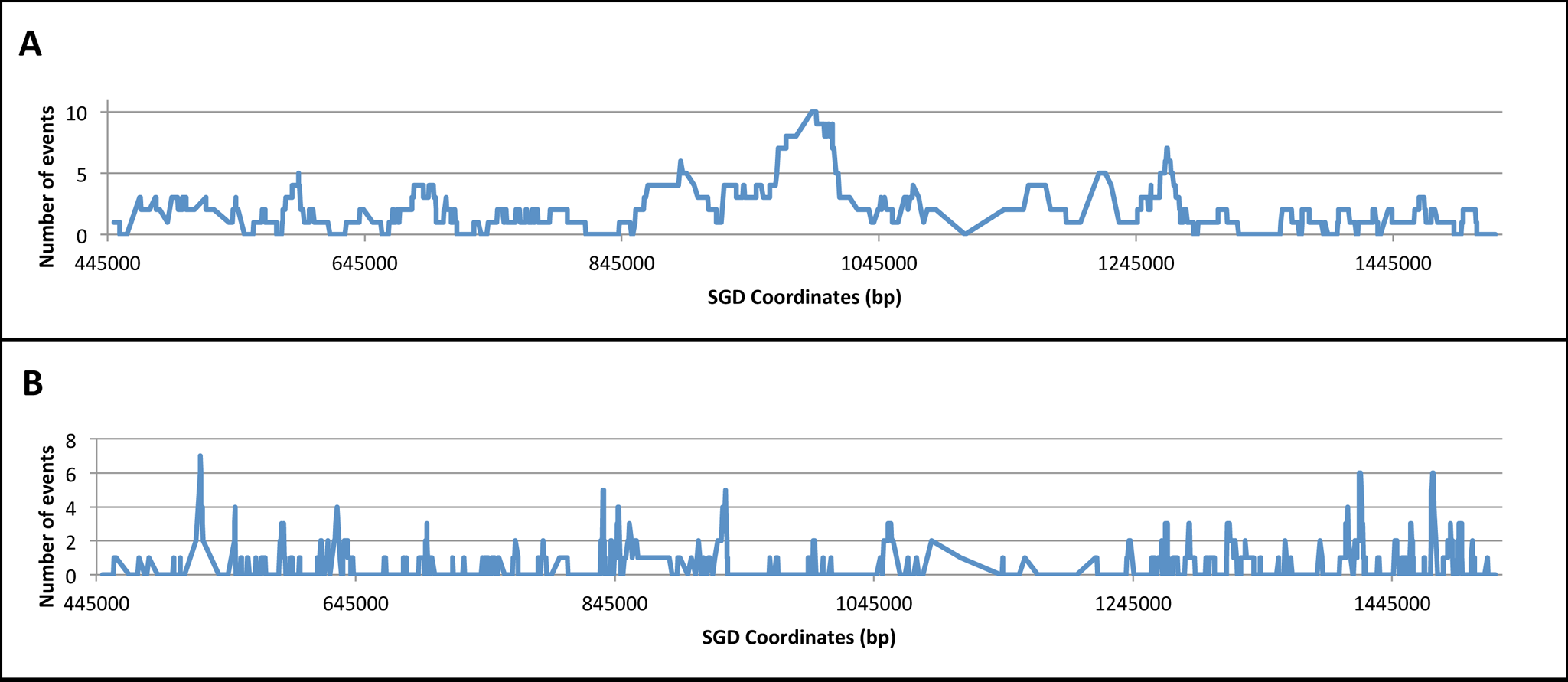

Supplement: Figure S6 — Comparison of mitotic and meiotic recombination maps on the right arm of chromosome IV. The Y-axis in the figure shows the number of times individual SNPs are included in a crossover-associated gene conversion. The Y-axis shows SGD coordinates on chromosome IV. A. Summary of our mapping of mitotic events in JSC25. B. Summary of the mapping of meiotic events in a closely-related diploid strain by [14]. (TIF) [file pgen.1003434.s006.tif]

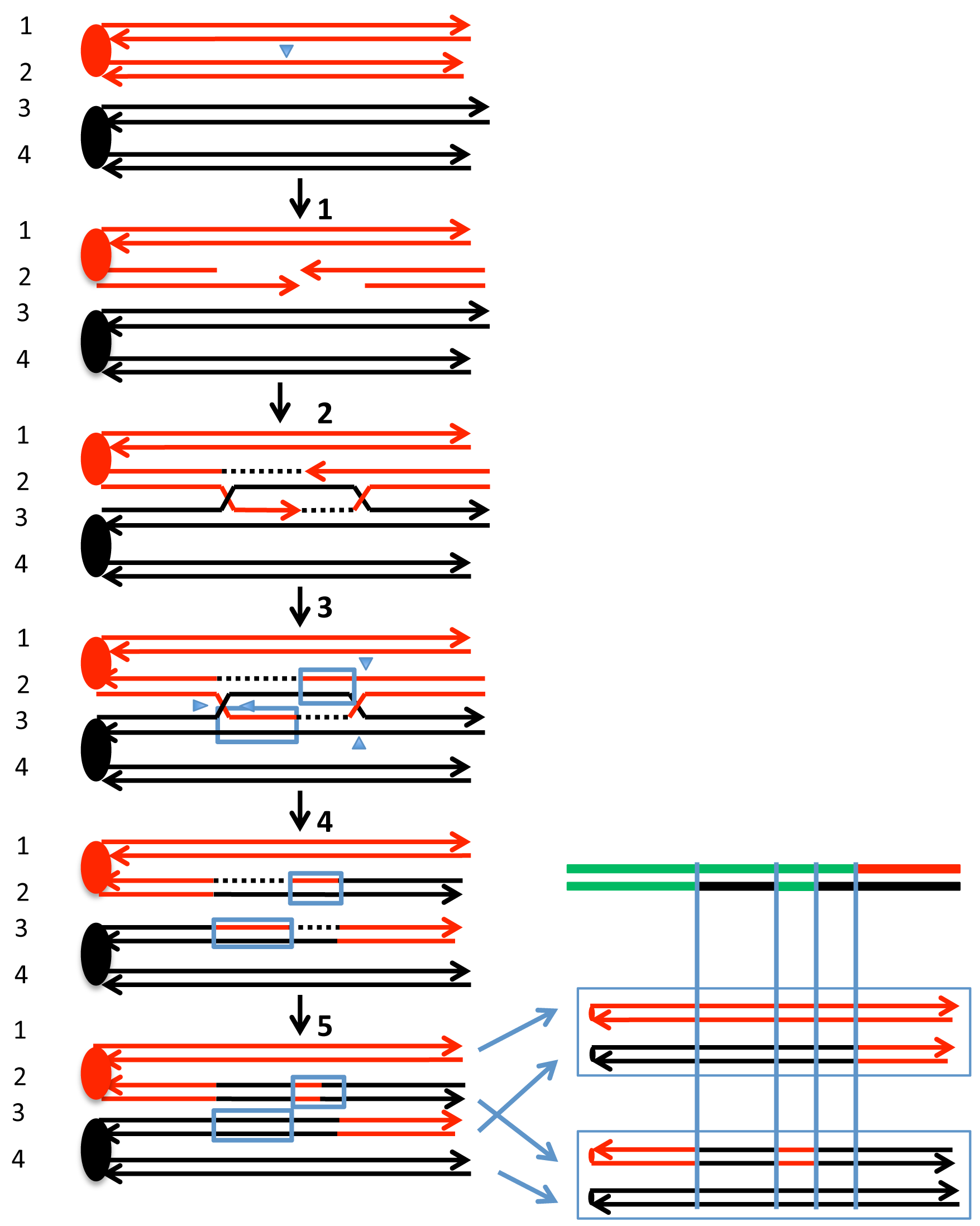

Supplement: Figure S7 — Generation of a complex conversion tract by “patchy” repair of mismatches in a heteroduplexes. This figure shows one mechanism for generating the complex gene conversion tract shown as the Class E2 event of Table S1. The homologs are depicted as double-stranded DNA molecules with the W303a-derived homolog shown as red lines and the YJM789-derived homolog shown as black lines. Recombination is initiated by a DSB on chromatid 2 in G2. The broken chromatid undergoes 5′ to 3′ resection (step 1), followed by strand invasion of the left end into chromatid 3 (step 2). DNA synthesis initiated by the broken end (shown as dotted lines), displaces one of the strands of chromatid 3, allowing second-end capture and DNA synthesis by the right hand broken end. The resulting double Holliday junction has two regions of heteroduplex (shown in blue rectangles), and is processed by cleaving the left and right junctions (cleavage sites shown by triangles) (step 3). Following junction resolution, the chromosome regions flanking the heteroduplexes are in the recombined configuration (step 4). In step 5, the mismatches within the two heteroduplexes are repaired. All of the mismatches in the heteroduplex on chromatid 3 are repaired in the same direction (duplicating YJM789-derived SNPs), whereas the heteroduplex on chromatid 4 undergoes “patchy” repair. Segregation of chromatids 1 and 3 into one cell and chromatids 2 and 4 into the other result in a crossover associated with the complex conversion tract depicted as Class E2 in Table S1. (TIF) [file pgen.1003434.s007.tif]
