# Supplementary material for: High-Resolution Mapping of Spontaneous Mitotic Recombination Hotspots on the 1.1 Mb Arm of Yeast Chromosome IV
Source: PLoS Genet. 2013 Apr 4;9(4):e1003434. doi: 10.1371/journal.pgen.1003434 (PMC3616911; doi:10.1371/journal.pgen.1003434)
Supplement: Table S2 — SGD coordinates for crossover and conversion transitions on chromosome IV. 1The classes of events are defined in Table S1. 2The lower case letters refer to transitions between heterozygous and homozygous regions as shown in Table S1. 3These numbers represent SGD coordinates of SNPs located on each side of the transition. It should be noted that these coordinates are based on SGD coordinates from Feb. 2010, and some of these coordinates may be different from those currently displayed in SGD. (DOCX) [file pgen.1003434.s009.docx]

Table S2. SGD coordinates for crossover and conversion transitions on chromosome IV.

| Sector | Event class^1^ | Transition label^2^ | Markers flanking transitions^3^ | |
| --- | --- | --- | --- | --- |
|  |  |  | Left | Right |
| 1RW | E8 | a | 1248863 | 1248966 |
|  |  | b | 1254353 | 1254489 |
|  |  | c | 1255048 | 1255087 |
|  |  | d | 1259633 | 1259877 |
|  |  | e | 1268296 | 1269558 |
|  |  | f | 1273350 | 1274404 |
| 2RW | E32 | a | 1373752 | 1374198 |
|  |  | b | 1375238 | 1379772 |
|  |  | c | 1380606 | 1385030 |
|  |  | d | 1385030 | 1386901 |
|  |  | e | 1387294 | 1388991 |
|  |  | f | 1388991 | 1390775 |
| 3RW | D1 | a | 1047033 | 1049553 |
|  |  | b | 1049553 | 1051286 |
|  |  | c | 1055397 | 1055663 |
| 4RW | E4 | a | 1157254 | 1161545 |
|  |  | b | 1161545 | 1171413 |
| 5RW | E33 | a | 959463 | 960043 |
|  |  | b | 964391 | 965740 |
|  |  | c | 1022402 | 1036052 |
|  |  | d | 1067771 | 1069072 |
|  |  | e | 1069072 | 1071135 |
|  |  | f | 1077836 | 1082836 |
| 6RW | E3 | a | 1190838 | 1216161 |
|  |  | b | 1225482 | 1231783 |
| 7RW | E3 | a | 1429103 | 1429226 |
|  |  | b | 1431061 | 1431782 |
| 9RW | B1 | a | 1275176 | 1275710 |
|  |  | b | 1275710 | 1276330 |
| 10RW | B2 | a | 1161545 | 1171413 |
|  |  | b | 1174705 | 1178332 |
| 11RW | D6 | a | 1308659 | 1308932 |
|  |  | b | 1310877 | 1311425 |
|  |  | c | 1311425 | 1311652 |
|  |  | d | 1315266 | 1315889 |
| 12RW | D4 | a | 1267082 | 1267721 |
|  |  | b | 1273350 | 1274404 |
|  |  | c | 1275176 | 1275710 |
| 13RW | E14 | a | 1438183 | 1441463 |
|  |  | b | 1470362 | 1470765 |
|  |  | c | 1476382 | 1476496 |
|  |  | d | 1476496 | 1477097 |
|  |  | e | 1477494 | 1479384 |
| 14RW | E21 | a | 1465228 | 1465378 |
|  |  | b | 1466345 | 1466614 |
|  |  | c | 1468543 | 1468701 |
|  |  | d | 1470362 | 1470765 |
| 15RW | D6 | a | 1267082 | 1267721 |
|  |  | b | 1273350 | 1274404 |
|  |  | c | 1278598 | 1279018 |
|  |  | d | 1289357 | 1289797 |
| 16RW | B2 | a | 1079615 | 1082836 |
|  |  | b | 1089446 | 1111969 |
| 18RW | E5 | a | 1040924 | 1044556 |
|  |  | b | 1047033 | 1049553 |
|  |  | c | 1049553 | 1051286 |
| 19RW | D1 | a | 1480252 | 1483459 |
|  |  | b | 1484403 | 1484580 |
|  |  | c | 1492290 | 1492550 |
| 20RW | E3 | a | 1067771 | 1069072 |
|  |  | b | 1071758 | 1075942 |
| 21RW | B2 | a | 1401638 | 1401858 |
|  |  | b | 1410413 | 1414518 |
| 23RW | A | a | 1161545 | 1171413 |
| 24RW | E12 | a | 1357190 | 1357597 |
|  |  | b | 1361012 | 1362046 |
|  |  | c | 1364731 | 1365816 |
|  |  | d | 1371117 | 1371846 |
| 25RW | A | a | 1201602 | 1216161 |
| 26RW | A | a | 1082836 | 1089446 |
| 27RW | D6 | a | 580548 | 580807 |
|  |  | b | 582114 | 582453 |
|  |  | c | 584836 | 585304 |
|  |  | d | 594021 | 595015 |
| 28RW | D4 | a | 1357597 | 1358761 |
|  |  | b | 1364731 | 1365816 |
|  |  | c | 1365816 | 1366726 |
| 29RW | E24 | a | 1292496 | 1293222 |
|  |  | b | 1297117 | 1297543 |
|  |  | c | 1304942 | 1305105 |
|  |  | d | 1310376 | 1310818 |
|  |  | e | 1313530 | 1313572 |
|  |  | f | 1317318 | 1317884 |
|  |  | g | 1320966 | 1321106 |
|  |  | h | 1324027 | 1324869 |
| 30RW | D6 | a | 1435418 | 1438183 |
|  |  | b | 1441931 | 1442062 |
|  |  | c | 1443817 | 1443889 |
|  |  | d | 1443889 | 1444237 |
| 31RW | A | a | 1111969 | 1142607 |
| 32RW | B1 | a | 1059384 | 1065071 |
|  |  | b | 1089446 | 1111969 |
| 33RW | D4 | a | 1402542 | 1402695 |
|  |  | b | 1411220 | 1414470 |
|  |  | c | 1415174 | 1416334 |
| 34RW | D2 | a | 1201602 | 1216161 |
|  |  | b | 1221046 | 1221462 |
|  |  | c | 1225482 | 1231783 |
| 35RW | B2 | a | 1256206 | 1256457 |
|  |  | b | 1256631 | 1257078 |
| 36RW | A | a | 1111969 | 1142607 |
| 37RW | E11 | a | 980838 | 992782 |
|  |  | b | 993347 | 993964 |
|  |  | c | 999490 | 999715 |
|  |  | d | 1002452 | 1003257 |
| 38RW | E15 | a | 512801 | 521609 |
|  |  | b | 526963 | 527305 |
|  |  | c | 527510 | 539130 |
|  |  | d | 545386 | 546290 |
|  |  | e | 546605 | 551364 |
| 39RW | A | a | 868904 | 889108 |
| 40RW | D6 | a | 964391 | 965740 |
|  |  | b | 975687 | 976249 |
|  |  | c | 1010105 | 1011955 |
|  |  | d | 1013909 | 1015206 |
| 41RW | A | a | 1190182 | 1216211 |
| 42RW | E34 | a | 868904 | 887030 |
|  |  | b | 976159 | 977414 |
|  |  | c | 980838 | 992782 |
|  |  | d | 996000 | 996527 |
| 43RW | D1 | a | 1190182 | 1216161 |
|  |  | b | 1217482 | 1217788 |
|  |  | c | 1231783 | 1235569 |
| 44RW | E8 | a | 1245782 | 1246274 |
|  |  | b | 1249203 | 1250136 |
|  |  | c | 1254154 | 1254293 |
| 45RW | E17 | a | 1417627 | 1418072 |
|  |  | b | 1420136 | 1420580 |
|  |  | c | 1422118 | 1422596 |
|  |  | d | 1430800 | 1431782 |
|  |  | e | 1433085 | 1433125 |
| 46RW | E1 | a | 889108 | 890618 |
|  |  | b | 890618 | 892648 |
|  |  | c | 895029 | 903910 |
| 47RW | E30 | a | 922159 | 924604 |
|  |  | b | 963792 | 965740 |
|  |  | c | 968451 | 969369 |
|  |  | d | 1028494 | 1036052 |
|  |  | e | 1037332 | 1039370 |
| 48RW | B1 | a | 774835 | 775273 |
|  |  | b | 779147 | 779952 |
| 49RW | D4 | a | 964391 | 965936 |
|  |  | b | 966485 | 966599 |
|  |  | c | 1010105 | 1012642 |
| 50RW | B1 | a | 1045316 | 1046711 |
|  |  | b | 1046711 | 1047033 |
| 51RW | B2 | a | 460205 | 469765 |
|  |  | b | 477827 | 482169 |
| 52RW | D1 | a | 907253 | 911910 |
|  |  | b | 916127 | 917134 |
|  |  | c | 918488 | 918591 |
| 53RW | A | a | 1161545 | 1171413 |
| 54RW | C2 | a | 855308 | 855878 |
|  |  | b | 911910 | 912446 |
| 55RW | D7 | a | 629580 | 630883 |
|  |  | b | 633870 | 633945 |
|  |  | c | 643241 | 643421 |
|  |  | d | 644066 | 651658 |
| 56RW | B2 | a | 1372403 | 1374333 |
|  |  | b | 1379772 | 1380606 |
| 57RW | B1 | a | 1059444 | 1065071 |
|  |  | b | 1066867 | 1069072 |
| 58RW | A | a | 1480252 | 1483459 |
| 59RW | C2 | a | 1161545 | 1171413 |
|  |  | b | 1201602 | 1216161 |
| 60RW | D5 | a | 768378 | 770295 |
|  |  | b | 770845 | 771197 |
|  |  | c | 774522 | 774716 |
|  |  | d | 776615 | 776665 |
| 61RW | D3 | a | 1111969 | 1142607 |
|  |  | b | 1146877 | 1147303 |
|  |  | c | 1161545 | 1171413 |
| 63RW | E8 | a | 1157254 | 1161545 |
|  |  | b | 1161545 | 1171413 |
|  |  | c | 1174705 | 1178332 |
| 64RW | D8 | a | 939092 | 939734 |
|  |  | b | 942797 | 943052 |
|  |  | c | 945141 | 945589 |
|  |  | d | 953272 | 955078 |
| 65RW | A | a | 1380606 | 1385030 |
| 66RW | D2 | a | 1111969 | 1142607 |
|  |  | b | 1185793 | 1186611 |
|  |  | c | 1190182 | 1216161 |
| 67RW | C2 | a | 980838 | 992782 |
|  |  | b | 1003836 | 1003935 |
| 68RW | D6 | a | 1461572 | 1461942 |
|  |  | b | 1465926 | 1466198 |
|  |  | c | 1468543 | 1468701 |
|  |  | d | 1469252 | 1469487 |
| 69RW | B2 | a | 1217788 | 1219699 |
|  |  | b | 1221462 | 1225482 |
| 70RW | D5 | a | 842451 | 843111 |
|  |  | b | 843111 | 843574 |
|  |  | c | 845115 | 845377 |
|  |  | d | 851194 | 852302 |
| 71RW | D4 | a | 1498068 | 1498508 |
|  |  | b | 1507062 | 1507520 |
|  |  | c | 1508989 | 1509699 |
| 72-1RW | D5 | a | 788875 | 789378 |
|  |  | b | 789378 | 790202 |
|  |  | c | 795523 | 795869 |
|  |  | d | 802606 | 802810 |
| 72-2RW | A | a | 1182080 | 1183438 |
| 73RW | B2 | a | 541971 | 542454 |
|  |  | b | 544627 | 544999 |
| 74RW | E12 | a | 739939 | 741160 |
|  |  | b | 747660 | 748528 |
|  |  | c | 748528 | 749244 |
|  |  | d | 752071 | 753192 |
| 75RW | A | a | 1346791 | 1349668 |
| 76RW | B2 | a | 491787 | 495183 |
|  |  | b | 495183 | 496923 |
| 77RW | E7 | a | 1201602 | 1216161 |
|  |  | b | 1220689 | 1221462 |
|  |  | c | 1221462 | 1225482 |
|  |  | d | 1225482 | 1231783 |
| 78RW | D3 | a | 729738 | 730063 |
|  |  | b | 730720 | 731914 |
|  |  | c | 733517 | 735346 |
| 79RW | D5 | a | 671494 | 672120 |
|  |  | b | 684381 | 684999 |
|  |  | c | 690253 | 690489 |
|  |  | d | 698494 | 699008 |
| 80RW | E16 | a | 1255964 | 1256206 |
|  |  | b | 1261433 | 1262162 |
|  |  | c | 1269558 | 1269689 |
|  |  | d | 1271391 | 1271682 |
|  |  | e | 1279397 | 1279784 |
| 81RW | E18 | a | 1231783 | 1235569 |
|  |  | b | 1240303 | 1242166 |
|  |  | c | 1251733 | 1252281 |
|  |  | d | 1265332 | 1266551 |
|  |  | e | 1269689 | 1269955 |
| 82RW | D8 | a | 558167 | 558882 |
|  |  | b | 563201 | 564349 |
|  |  | c | 571796 | 572478 |
|  |  | d | 576294 | 578154 |
| 83RW | B1 | a | 1391617 | 1392166 |
|  |  | b | 1392166 | 1394070 |
| 84RW | D5 | a | 1281755 | 1282224 |
|  |  | b | 1282790 | 1283368 |
|  |  | c | 1283368 | 1283747 |
|  |  | d | 1284092 | 1284918 |
| 85RW | B1 | a | 1071233 | 1071758 |
|  |  | b | 1075942 | 1077702 |
| 86RW | B1 | a |  | 449787 |
|  |  | b | 454159 | 454186 |
| 87RW | C1 | a | 713743 | 714477 |
|  |  | b | 715012 | 715335 |
| 88RW | C2 | a | 1017595 | 1022402 |
|  |  | b | 1022402 | 1028494 |
| 89RW | E22 | a | 460205 | 469765 |
|  |  | b | 471247 | 476888 |
|  |  | c | 477827 | 482169 |
|  |  | d | 482169 | 482635 |
|  |  | e | 482635 | 482940 |
| 90RW | E31 | a | 682940 | 683460 |
|  |  | b | 686861 | 687261 |
|  |  | c | 698254 | 698333 |
|  |  | d | 699796 | 699924 |
|  |  | e | 700648 | 700759 |
| 91RW | D6 | a | 581168 | 581504 |
|  |  | b | 582114 | 582453 |
|  |  | c | 585541 | 586687 |
|  |  | d | 593737 | 594021 |
| 92RW | D6 | a | 1263246 | 1264102 |
|  |  | b | 1265332 | 1266551 |
|  |  | c | 1267721 | 1267763 |
|  |  | d | 1271793 | 1271980 |
| 93RW | D3 | a | 1263246 | 1264102 |
|  |  | b | 1266671 | 1266933 |
|  |  | c | 1267082 | 1267721 |
| 94RW | A | a | 1111969 | 1142607 |
| 95RW | B1 | a | 668612 | 669986 |
|  |  | b | 670622 | 671011 |
| 96RW | E23 | a | 694543 | 694817 |
|  |  | b | 698211 | 698254 |
|  |  | c | 700759 | 701050 |
|  |  | d | 703507 | 703836 |
|  |  | e | 706589 | 706908 |
|  |  | f | 707328 | 708625 |
|  |  | g | 708625 | 709196 |
| 97RW | D8 | a | 861704 | 862566 |
|  |  | b | 863830 | 865277 |
|  |  | c | 893739 | 894438 |
|  |  | d | 909432 | 911910 |
| 98RW | E6 | a | 544266 | 544627 |
|  |  | b | 545033 | 545123 |
|  |  | c | 546605 | 551364 |
| 99RW | B2 | a | 889108 | 890618 |
|  |  | b | 890618 | 892648 |
| 100RW | D5 | a | 747660 | 748528 |
|  |  | b | 751098 | 752071 |
|  |  | c | 754411 | 755087 |
|  |  | d | 755087 | 755446 |
| 101RW | B2 | a | 1499309 | 1499476 |
|  |  | b | 1510193 | 1510396 |
| 102RW | D2 | a | 593628 | 593737 |
|  |  | b | 596803 | 597003 |
|  |  | c | 597003 | 598170 |
| 103RW | E19 | a | 949439 | 949782 |
|  |  | b | 951654 | 952255 |
|  |  | c | 952316 | 953272 |
|  |  | d | 970231 | 970419 |
|  |  | e | 1000365 | 1001602 |
|  |  | f | 1006070 | 1006514 |
| 104RW | E20 | a | 868904 | 887030 |
|  |  | b | 895029 | 901094 |
|  |  | c | 901094 | 907253 |
| 105RW | A | a | 460205 | 469765 |
| 106RW | E3 | a | 477827 | 482169 |
|  |  | b | 521609 | 524793 |
| 107RW | D6 | a | 972912 | 973113 |
|  |  | b | 976249 | 977414 |
|  |  | c | 1007002 | 1007144 |
|  |  | d | 1008785 | 1008829 |
| 108RW | E9 | a | 777015 | 777375 |
|  |  | b | 794043 | 794806 |
|  |  | c | 796161 | 802539 |
| 109RW | B1 | a | 491787 | 495183 |
|  |  | b | 495183 | 496923 |
| 110RW | B2 | a | 460205 | 469765 |
|  |  | b | 470717 | 471226 |
| 111RW | C2 | a | 863830 | 865277 |
|  |  | b | 868904 | 887030 |
| 112RW | D5 | a | 663372 | 663847 |
|  |  | b | 674798 | 675271 |
|  |  | c | 696034 | 696092 |
|  |  | d | 700236 | 700426 |
| 113RW | B1 | a | 666145 | 666933 |
|  |  | b | 667323 | 667565 |
| 114RW | B1 | a | 564732 | 565232 |
|  |  | b | 567351 | 567792 |
| 115RW | E13 | a | 853434 | 855165 |
|  |  | b | 863830 | 865277 |
|  |  | c | 868013 | 868562 |
|  |  | d | 868904 | 887030 |
| 116RW | D5 | a | 796161 | 802110 |
|  |  | b | 808984 | 809854 |
|  |  | c | 811980 | 812466 |
|  |  | d | 816605 | 817112 |
| 117RW | D6 | a | 966213 | 966485 |
|  |  | b | 980838 | 992782 |
|  |  | c | 1004465 | 1005641 |
|  |  | d | 1013370 | 1013909 |
| 118RW | A | a | 796161 | 802110 |
| 119RW | D8 | a | 495183 | 496923 |
|  |  | b | 509817 | 512538 |
|  |  | c | 524793 | 525397 |
|  |  | d | 527510 | 539083 |
| 120RW | D6 | a | 754032 | 754057 |
|  |  | b | 755446 | 755533 |
|  |  | c | 761361 | 761909 |
|  |  | d | 765384 | 766033 |
| 121RW | C2 | a | 706018 | 706589 |
|  |  | b | 712108 | 712459 |
| 122RW | A | a | 796161 | 802110 |
| 124RW | E10 | a | 1004465 | 1005641 |
|  |  | b | 1007819 | 1008785 |
|  |  | c | 1008785 | 1008829 |
|  |  | d | 1010105 | 1011921 |
| 128RW | D6 | a | 922159 | 924604 |
|  |  | b | 930041 | 930998 |
|  |  | c | 931361 | 932198 |
|  |  | d | 940065 | 940980 |
| 129RW | B1 | a | 761909 | 762882 |
|  |  | b | 771197 | 772684 |
| 130RW | E26 | a | 1474547 | 1474663 |
|  |  | b | 1474866 | 1475232 |
|  |  | c | 1477494 | 1479384 |
|  |  | d | 1480252 | 1483459 |
| 131RW | D7 | a | 1003257 | 1003678 |
|  |  | b | 1003678 | 1003797 |
|  |  | c | 1007819 | 1008785 |
|  |  | d | 1008785 | 1008829 |
| 133RW | E28 | a | 587919 | 588369 |
|  |  | b | 588547 | 588694 |
|  |  | c | 593197 | 593449 |
|  |  | d | 594021 | 595514 |
|  |  | e | 597003 | 598170 |
|  |  | f | 604773 | 605544 |
| 136RW | E2 | a | 601671 | 601816 |
|  |  | b | 604427 | 604773 |
|  |  | c | 611081 | 611611 |
|  |  | d | 616610 | 617827 |
| 139RW | D6 | a | 1007819 | 1008785 |
|  |  | b | 1011921 | 1012642 |
|  |  | c | 1015206 | 1017595 |
|  |  | d | 1017595 | 1028494 |
| 140RW | D8 | a | 681904 | 681933 |
|  |  | b | 681933 | 682289 |
|  |  | c | 686024 | 686695 |
|  |  | d | 690515 | 690737 |
| 142RW | D8 | a | 583189 | 583420 |
|  |  | b | 584836 | 585304 |
|  |  | c | 588694 | 589427 |
|  |  | d | 594021 | 595015 |
| 143RW | E8 | a | 1268296 | 1268882 |
|  |  | b | 1272145 | 1272729 |
|  |  | c | 1278598 | 1279018 |
| 144RW | E25 | a | 1190182 | 1216161 |
|  |  | b | 1217163 | 1217192 |
|  |  | c | 1217192 | 1217482 |
|  |  | d | 1217788 | 1219699 |
| 145RW | B1 | a | 640958 | 641270 |
|  |  | b | 657097 | 658422 |
| 146RW | B2 | a | 922159 | 924604 |
|  |  | b | 934165 | 934343 |
| 147RW | A | a | 853434 | 855165 |
| 149RW | E27 | a | 707328 | 708625 |
|  |  | b | 711298 | 711895 |
|  |  | c | 712684 | 712843 |
|  |  | d | 713491 | 714477 |
|  |  | e | 716627 | 717363 |
| 150RW | D4 | a | 476888 | 482169 |
|  |  | b | 482635 | 482940 |
|  |  | c | 485707 | 491787 |
| 153RW | B1 | a | 495183 | 496923 |
|  |  | b | 499309 | 499633 |
| 154RW | B1 | a | 502953 | 503234 |
|  |  | b | 505869 | 507475 |
| 156RW | A | a | 485707 | 491787 |
