# Supplementary material for: High-Resolution Mapping of Spontaneous Mitotic Recombination Hotspots on the 1.1 Mb Arm of Yeast Chromosome IV
Source: PLoS Genet. 2013 Apr 4;9(4):e1003434. doi: 10.1371/journal.pgen.1003434 (PMC3616911; doi:10.1371/journal.pgen.1003434)
Supplement: Table S4 — Chromosome elements examined for their representation in mitotic gene conversion tracts. 1For most of the listed elements, we examined the number of such elements between CEN4 and the markers near the right telomere used to diagnose crossovers. 2Palindromes that were at least 16 bp are counted. 3The repeats examined were in the size range of 2 to 213 bp, with a minimum repeat tract of 24 bp. 4This database is accessible at the Website: https://tandem.bu.edu/cgi-bin/trdb/trdb.exe 5The minimal number of repeats/tract was 8. 6The G4 motifs used in this analysis were four tracts of 3 G's separated by spacers of less than 25 bp. 7Transcription levels were measured for 451 of the 565 ORFs on the right arm of chromosome IV in [19]. Most of these genes were single-copy sequences, since measuring transcription from repeated genes with very similar sequences is difficult. We identified the twenty single-copy genes in this region with the highest level of transcription. 8Peaks of accumulation of gamma-H2AX within the genome were mapped; such regions are often associated with DNA damage [20]. 9Rrm3p is a helicase involved in promoting replication through replication-pause sites [21]. 10We identified the locations of the 17 longest intergenic regions of a total of 554 on the right arm of chromosome IV. 11ARS elements were identified in which the genes flanking the ARS have transcripts that converge on the ARS. (DOCX) [file pgen.1003434.s011.docx]

Table S4. Chromosome elements examined for their representation in mitotic gene conversion tracts.

| **Chromosome element (# of each element present on the right arm of chromosome IV)^1^** | **Description or reference** |
| --- | --- |
| Palindromes^2^ (46) | ([15]; Z. Zgaga, personal communication) |
| Tandem repeats^3^ (97) | Tandem-repeat database (TRDB)^4^ [16] |
| Triplet repeats^5^ (10) | Triplet repeats as identified in the TRDB |
| G4 DNA^6^ (34) | Dataset S1 in [17] |
| tRNA genes (23) | Saccharomyces Genome Database (SGD) |
| Short (150 bp) intergenic regions (61) | Saccharomyces Genome Database (SGD) |
| Intron-containing genes (24) | Saccharomyces Genome Database (SGD) |
| ARS elements (28) | Saccharomyces Genome Database (SGD) |
| Early- and middle-firing ARS elements (10) | Table S1 in [18] |
| Long terminal repeats (LTRs) (33) | Saccharomyces Genome Database (SGD) |
| Long terminal repeats (> 1 kb) from a Ty element (11) | Saccharomyces Genome Database (SGD) |
| Highly-transcribed genes^7^ (20) | Table S4 in [19] |
| Gamma-H2AX peaks^8^ (38) | Supp. Data 1 in [20] |
| Rrm3p pause sites^9^ (6) | Supp. Table 7 in [21] |
| Long intergenic regions^10^ (17) | Saccharomyces Genome Database (SGD) |
| Replication-termination regions (TERs) (3) | Table S2 in [18] |
| ARS elements flanked by opposing transcripts^11^ (12) | Saccharomyces Genome Database (SGD) |
| Ty elements (8) | Saccharomyces Genome Database (SGD) |
