# Supplementary material for: High-Resolution Mapping of Spontaneous Mitotic Recombination Hotspots on the 1.1 Mb Arm of Yeast Chromosome IV
Source: PLoS Genet. 2013 Apr 4;9(4):e1003434. doi: 10.1371/journal.pgen.1003434 (PMC3616911; doi:10.1371/journal.pgen.1003434)
Supplement: Table S5 — Chromosome elements that are over-represented in crossover-associated gene conversion tracts in JSC25. 1As described in the text, we used four related methods of analysis to determine whether various structure/sequence chromosome motifs (listed in Table S4) were over-represented in gene conversion tracts associated with crossovers on chromosome IV. 2The data examined by Methods 1-4 were: JSC25 all (G1 and G2 events initiated on either W303a- and YJM789-derived homologs), JSC25 G1 (G1 events initiated on either W303a- and YJM789-derived homologs), JSC25 G2 (G2 events initiated on either W303a- and YJM789-derived homologs), W303a all (G1 and G2 events initiated on the W303-derived homologs), and YJM789 all (G1 and G2 events initiated on the YJM789-derived homologs). 3Rrm3p is a helicase that promotes replication fork progression through regions at which the forks are paused [21]. (DOCX) [file pgen.1003434.s012.docx]

Table S5. Chromosome elements that are over-represented in crossover-associated gene conversion tracts in JSC25.^1^

| Element | Data^2^ | Method of analysis | p-value |
| --- | --- | --- | --- |
| Long terminal repeats (deltas) | JSC25 all | 1 | 1.55E-07 |
| Long terminal repeats (deltas) | JSC25 all | 3 | 1.04E-11 |
| Long terminal repeats (deltas) | JSC25 G1 | 1 | 6.06E-09 |
| Long terminal repeats (deltas) | JSC25 G1 | 3 | 1.44E-15 |
| Long terminal repeats (deltas) | W303a all | 1 | 1.28E-06 |
| Long terminal repeats (deltas) | W303a all | 3 | 7.31E-14 |
| Ty elements | JSC25 all | 1 | 1.39E-05 |
| Ty elements | JSC25 all | 3 | 2.13E-06 |
| Ty elements | JSC25 G1 | 1 | 1.70E-06 |
| Ty elements | JSC25 G1 | 3 | 4.45E-08 |
| Ty elements | W303a all | 1 | 3.44E-06 |
| Ty elements | W303a all | 3 | 1.28E-07 |
| tRNA genes | JSC25 all | 3 | 0.011 |
| tRNA genes | JSC25 G1 | 3 | 0.0019 |
| tRNA genes | W303a all | 3 | 0.002 |
| G4 motifs | JSC25 G1 | 4 | 3.72E-04 |
| G4 motifs | YJM789 all | 4 | 4.27E-04 |
| Replication termination region | JSC25 G2 | 1 | 2.77E-04 |
| Replication termination region | JSC25 G2 | 2 | 3.82E-04 |
| Replication termination region | JSC25 G2 | 3 | 1.67E-04 |
| Replication termination region | JSC25 G2 | 4 | 1.98E-04 |
| Rrm3p pause sites^3^ | YJM789 all | 2 | 0.0024 |
