# Supplementary material for: High-Resolution Mapping of Spontaneous Mitotic Recombination Hotspots on the 1.1 Mb Arm of Yeast Chromosome IV
Source: PLoS Genet. 2013 Apr 4;9(4):e1003434. doi: 10.1371/journal.pgen.1003434 (PMC3616911; doi:10.1371/journal.pgen.1003434)
Supplement: Table S6 — Chromosome elements that are under-represented in crossover-associated gene conversion tracts in JSC25. 1As described in the text, we used four related methods of analysis to determine whether various structure/sequence chromosome motifs (listed in Table S4) were under-represented in gene conversion tracts associated with crossovers on chromosome IV. 2The data examined by Methods 1–4 were: JSC25 all (G1 and G2 events initiated on either W303a- and YJM789-derived homologs), JSC25 G1 (G1 events initiated on either W303a- and YJM789-derived homologs), JSC25 G2 (G2 events initiated on either W303a- and YJM789-derived homologs), W303a all (G1 and G2 events initiated on the W303-derived homologs), and YJM789 all (G1 and G2 events initiated on the YJM789-derived homologs). (DOCX) [file pgen.1003434.s013.docx]

Table S6. Chromosome elements that are under-represented in crossover-associated gene conversion tracts in JSC25.^1^

| **Element** | **Data^2^** | **Method of analysis** | **p-Value** |
| --- | --- | --- | --- |
| Long terminal repeats | JSC25 G1 | 2 | 1.65E-03 |
| Tandem repeats | JSC25 all | 1 | 7.51E-05 |
| Tandem repeats |  | 3 | 1.15E-06 |
| Tandem repeats | JSC25 G1 | 1 | 2.39E-04 |
| Tandem repeats |  | 3 | 6.24E-06 |
| Tandem repeats | W303a all | 1 | 6.53E-03 |
| Tandem repeats |  | 3 | 2.95E-03 |
| Tandem repeats | YJM789 all | 3 | 7.10E-04 |
| Short intergenic regions | JSC25 all | 1 | 1.12E-03 |
| Short intergenic regions |  | 3 | 5.22E-05 |
| Short intergenic regions | JSC25 G1 | 1 | 7.15E-03 |
| Short intergenic regions |  | 3 | 2.80E-04 |
| Short intergenic regions | W303a all | 1 | 4.87E-03 |
| Short intergenic regions |  | 3 | 4.26E-04 |
| Highly-transcribed genes | JSC25 all | 3 | 7.69E-03 |
| Highly-transcribed genes | JSC25 G1 | 3 | 5.17E-03 |
