# Supplementary material for: High-Resolution Mapping of Spontaneous Mitotic Recombination Hotspots on the 1.1 Mb Arm of Yeast Chromosome IV
Source: PLoS Genet. 2013 Apr 4;9(4):e1003434. doi: 10.1371/journal.pgen.1003434 (PMC3616911; doi:10.1371/journal.pgen.1003434)
Supplement: Table S7 — Strain list and strain constructions. 1 Standard gene nomenclature is used with a few exceptions. An insertion unaccompanied by a deletion is indicated by a double colon; a replacement is indicated by a Δ, followed by a double colon and the name of the replacing gene. If an insertion is placed within a genomic sequence unassociated with a gene name, we indicate the position of the centromere-proximal base followed by a double colon and the name of the inserted gene. For example, IV10132176::URA3 indicates that the URA3 gene was inserted between bases 10132176 and 10132177 on chromosome IV. For diploid genotypes, the genotype of the W303a-derived haploid is shown above the diagonal and the genotype of the YJM789-derived haploid is shown below the diagonal. The HS4 hotspot (inverted Ty elements) is missing on the YJM789-derived homology; the nomenclature used to describe the missing Ty elements in the genotypes above is: ydrwTy2-3Δ. (DOCX) [file pgen.1003434.s014.docx]

Table S7. Strain list and strain constructions^1^.

| **Strain** | **Genotype^1^** | **Reference/construction** |
| --- | --- | --- |
| PSL4 | *MATα ade2-1 ura3 gal2 ho::hisG* | Isogenic with YJM789 [3] |
| W303a | *MATa leu2-3,112 his3-11,15 ura3-1 ade2-1 trp1-1 can1-100 rad5* | [22] |
| W1588-4c | *MATa leu2-3,112 his3-11,15 ura3-1 ade2-1 trp1-1 can1-100 RAD5* | Isogenic with W303a [23] |
| JAY291 | *MATa* wild-type haploid | [24] |
| JSC10-1 | *MATa leu2-3,112 his3-11,15 ura3-1 ade2-1 trp1-1 can1-100Δ::NAT RAD5* | W1588-4c with replacement of *can1-100* with *NAT.* Isogenic with W303a. |
| JSC11-1 | *MATa leu2-3,112 his3-11,15 ura3-1 ade2-1 trp1-1 can1-100 V31539::KANMX* | W303a with *KANMX* inserted between bases V31539 and V31540. |
| JSC12-1 | *MATa leu2-3,112, his3-11,15 ura3-1 ade2-1 trp1-1 can1-100Δ::NAT RAD5 IV1510386::KANMX-can1-100* | JSC10-1 transformed with *KANMX-can1-100* cassette inserted between bases IV1510386 and 1510387. Isogenic with W303a. |
| JSC19-1 | *MATα ade2-1 ura3 gal2 ho::hisG CAN1Δ::NAT* | PSL4 with *CAN1* replaced with *NAT.* Isogenic with YJM789. |
| JSC21-1 | *MATα ade2-1 ura3 gal2 ho::hisG CAN1Δ::NAT IV1510386::SUP4-o* | JSC19-1 with insertion of *SUP4-o* at IV1510386. Isogenic with YJM789. |
| JSC22-1 | *MATa/MATα leu2-3,112/LEU2 his3-11,15/HIS3 ura3-1 /ura3 GAL2/gal2 ade2-1/ade2-1 trp1-1/TRP1 can1-100Δ::NAT/ CAN1Δ::NAT RAD5/RAD5 IV1510386::KANMX-can1-100/IVI1510386::SUP4-o* | Cross of JSC12-1 and JSC21-1. |
| JSC25-1 | *MATa/MATα::HYG leu2-3,112/LEU2 his3-11,15/HIS3 ura3-1 /ura3 GAL2/gal2 ade2-1/ade2-1 trp1-1/TRP1 can1-100Δ::NAT/ CAN1Δ::NAT RAD5/RAD5 IV1510386::KANMX-can1-100/IVI1510386::SUP4-o* | JSC22-1 with *MATα* replaced with *HYG.* |
| JSC52-1 | *MATa leu2-3,112 his3-11,15 ura3-1 ade2-1 trp1-1 can1-100 RAD5 IV1013217::URA3* | W1588-4c with *URA3* inserted centromere-proximal to HS4 at position IV1013217. Isogenic with W303a. |
| JSC54-1 | *MATa leu2-3,112 his3-11,15 ura3-1 ade2-1 trp1-1 can1-100 RAD5 IV957578::HYG IV1013217::URA3* | JSC52-1 with *HYG* inserted centromere-distal to HS4 at position IV957578. Isogenic with W303a. |
| JSC57-1 | *MATa leu2-3,112 his3-11,15 ura3-1 ade2-1 trp1-1 can1-100 RAD5 IV957578::HYG IV1013217::URA3 YDRWTy2-3Δ::KANMX* | JSC54-1 with a replacement of *YDRWTy2-3* with *KANMX*. Isogenic with W303a. |
| JSC58-2 | *MATa leu2-3,112 his3-11,15 ura3-1 ade2-1 trp1-1 can1-100 RAD5 IV957578::HYG IV1013217::URA3 YDRWdelta19Δ:: KANMX* | JSC54-1 with a replacement of *YDRWdelta19* with *KANMX*. Isogenic with W303a. |
| JSC59-2 | *MATa leu2-3,112 his3-11,15 ura3-1 ade2-1 trp1-1 can1-100 RAD5 IV957578::HYG IV1013217::URA3 IV987138:: KANMX* | JSC54-1 with *KANMX* inserted between the two Ty elements of HS4 at position IV987138. Isogenic with W303a. |
| JSC67-1 | *MATa/MATα ade2-1/ade2-1 ura3-1/ura3 GAL2/gal2 ho/ho::hisG can1-100/CAN1 leu2-3,112/ LEU2 his3-11,15/ HIS3 trp1-1/TRP1 IV957578::HYG/ IV957578 IV10132176::URA3/IV1013217* | Cross of JSC54-1 and PSL4 |
| JSC68-1 | *MATa/MATα ade2-1/ade2-1 ura3-1/ura3 GAL2/gal2 ho/ho::hisG can1-100/CAN1 leu2-3,112/ LEU2 his3-11,15/ HIS3 trp1-1/TRP1 IV957578::HYG/ IV957578 IV10132176::URA3/IV1013217 YDRWTy2-3Δ::KANMX/ydrwTy2-3Δ* | Cross of JSC57-1 and PSL4 |
| JSC70-3 | *MATa/MATα ade2-1/ade2-1 ura3-1/ura3 GAL2/gal2 ho/ho::hisG can1-100/CAN1 leu2-3,112/ LEU2 his3-11,15/ HIS3 trp1-1/TRP1 IV957578::HYG/ IV957578 IV10132176::URA3/IV1013217 IV987138:: KANMX/IV987138* | Cross of JSC59-2 and PSL4 |
| JSC71-1 | *MATa/MATα::NAT ade2-1/ade2-1 ura3-1/ura3 GAL2/gal2 ho/ho::hisG can1-100/CAN1 leu2-3,112/ LEU2 his3-11,15/ HIS3 trp1-1/TRP1 IV957578::HYG/ IV957578 IV10132176::URA3/IV1013217* | *MATα * derivative of JSC67-1 |
| JSC73-2 | *MATa/MATαΔ::NAT ade2-1/ade2-1 ura3-1/ura3 GAL2/gal2 ho/ho::hisG can1-100/CAN1 leu2-3,112/ LEU2 his3-11,15/ HIS3 trp1-1/TRP1 IV957578::HYG/ IV957578 IV10132176::URA3/IV1013217 YDRWTy2-3::KANMX/ydrwTy2-3Δ* | *MATα * derivative of JSC68-1 |
| JSC74-1 | *MATa/MATα::NAT ade2-1/ade2-1 ura3-1/ura3 GAL2/gal2 ho/ho::hisG can1-100/CAN1 leu2-3,112/ LEU2 his3-11,15/ HIS3 trp1-1/TRP1 IV957578::HYG/ IV957578 IV10132176::URA3/IV1013217 IV987138:: KANMX/IV987138* | *MATα * derivative of JSC70-3*.* |
| JSC75-1 | *MATa/MATα ade2-1/ade2-1 ura3-1/ura3 GAL2/gal2 ho/ho::hisG can1-100/CAN1 leu2-3,112/ LEU2 his3-11,15/ HIS3 trp1-1/TRP1 IV957578::HYG/ IV957578 IV10132176::URA3/IV1013217 YDRWdelta19:: KANMX/ydrwdelta19Δ* | Cross of JSC58-2 and PSL4. |
| JSC77-1 | *MATa/MATα::NAT ade2-1/ade2-1 ura3-1/ura3 GAL2/gal2 ho/ho::hisG can1-100/CAN1 leu2-3,112/ LEU2 his3-11,15/ HIS3 trp1-1/TRP1 IV957578::HYG/ IV957578 IV10132176::URA3/IV1013217 YDRWdelta19:: KANMX/ydrwdelta19Δ* | *MATα * derivative of JSC75-1*.* |
