# Supplementary material for: High-Resolution Mapping of Spontaneous Mitotic Recombination Hotspots on the 1.1 Mb Arm of Yeast Chromosome IV
Source: PLoS Genet. 2013 Apr 4;9(4):e1003434. doi: 10.1371/journal.pgen.1003434 (PMC3616911; doi:10.1371/journal.pgen.1003434)
Supplement: Table S8 — Names and sequences of primers used in strain constructions and genome analysis. 1Primer sequences are written 5′ to 3′. (DOCX) [file pgen.1003434.s015.docx]

Table S8. Names and sequences of primers used in strain constructions and genome analysis.

| **Primer name** | **Primer sequence^1^** |
| --- | --- |
| V31539::DR F | AGGTCTGAAGGAGTTTCAAATGCTTCTACTCCGTCTGCTTTCTTTTCGGGACGTACGCTGCAGGTCGAC |
| W CAN1 KO R | ACTGGCAAGTGCGTATAAATTAAACCTATTTCTTTATCATCATATTTACTTATCGATGAATTCGAGCTCG |
| CAN1-500 F | GAGTTCTGCCCTTGGCTTCC |
| W2 CAN1 KO R | GGATTTGGCGTGGATGAAGG |
| V31539::DR R | ATAATCTCAGTATATTTATTTTTATCGATGATTCACCACAACAATCTTGCTATCGATGAATTCGAGCTCG |
| IV1510386::can1-100 F | CATACGTTATGCACTTCATTCTTCTTGTCGGTTTGATAACAGCAGAATCTACGTACGCTGCAGGTCGAC |
| IV1510386::can1-100 R | GCGTTTTCGAGGTATGGCTTCTGCCGGGCTAACGTTCAAATTAAAGGAACTTATATCTTTAACAGATTCCAA |
| YJMIV::SUP4-o F | CATACGTTATGCACTTCATTCTTTTTGTCGGTTTGATACCAGCAGAATCTAGGATCCGGGACCGGATAAT |
| YJMIV::SUP4-o R | GCGTTTTCGAGGTATGGCTTCTGTAGGGTTAACGCTCAAATTAAAGGAACTGGATCCGGAATTCTTGAAAG |
| IV 980403 F | TTGAACATGGTCGCGACAGC |
| Ty2 R | GCCGTGCTGTTGGTACTGTCC |
| Ty1.2 R | TTGAGGAGAGGCATGATGG |
| IV 993256 F | CTGGTGCTAACCGCTTGTGC |
| RE HS4 URA3 F | TTTATATAGATAAACAAACTTGCAGGACAGATAGTTAAGCGTCTATATCATAATGTGGCTGTGGTTTCAGG |
| RE HS4 URA3 R | TCTTTTTTGCCTTTTATCATTTTTGTACTTTTTTCTTCGCTTAAAATACACAGATTCCCGGGTAATAACTG |
| RE HS4 HYG EXT F | CACAAGCACTTCTTGCGAGTCC |
| RE HS4 HYG EXT R | GGAGTCTCAATTCAGGGATCTGG |
| HS4 Ty2-3::KAN F | GTTAATAAAACACACATGCAACTTGTTGGAATAAAAATCCACTATCGTCTTCGTACGCTGCAGGTCGAC |
| HS4 Ty2-3::KAN R | TTATCTAATTACCCACATATATCTCATAACTATTAGTTGATAGACGTGTAGATCGATGAATTCGAGCTCG |
| HS4 DELTA19::KAN R | CTTGATTTGATGGGACTTCCTTAGAAGTAACCGAAGCAGCGGCGCTACCATATCGATGAATTCGAGCTCG |
| HS4 SPACER:: KAN F | AATGGAATCCCAACAATTACATCAAAATCCACATTCTCTACACGTCTATCACGTACGCTGCAGGTCGAC |
| HS4 SPACER::KAN R | GGAATCCCAACAATTATCTAATTACCCACATATATCTCATAACTATTAGTTATCGATGAATTCGAGCTCG |
