# Supplementary material for: High-Resolution Mapping of Spontaneous Mitotic Recombination Hotspots on the 1.1 Mb Arm of Yeast Chromosome IV
Source: PLoS Genet. 2013 Apr 4;9(4):e1003434. doi: 10.1371/journal.pgen.1003434 (PMC3616911; doi:10.1371/journal.pgen.1003434)
Supplement: Text S1 — Supplemental materials and methods and discussions. (DOCX) [file pgen.1003434.s017.docx]

## Text S1

**High-Resolution Mapping of Spontaneous Mitotic Recombination Hotspots on the 1.1 Mb Arm of Yeast Chromosome IV**

Jordan St Charles and Thomas D. Petes

### Supplemental materials and methods

#### Strain construction

In order to examine mitotic reciprocal crossovers on the right arm of chromosome IV, we used the color screening system that is described in the Results section. This system involved a W303a-YJM789 hybrid strain with an insertion of a *can1-100-KANMX* cassette near the right telomere of chromosome IV (SGD coordinate 1151 kb) on the W303a-derived homolog and insertion of *SUP4-o* at the allelic position on the YJM789-derived homolog. All strains used in this study are listed in Table S7, and all primers used for strain construction are listed in Table S8, unless stated otherwise.

In our previous study of recombination on chromosome V, we selected crossovers in diploids in which the *can1-100* mutation (a nonsense-suppressible allele) was located near the telomere of chromosome V on one homolog and the *SUP4-o* gene was located at an allelic position on the other homolog, replacing the *CAN1* gene [1]; the diploid was also homozygous for the *ade2-1* mutation. Crossovers on chromosome V produced canavanine-resistant red/white sectored colonies. In order to generate a similar system for chromosome IV, we deleted the *CAN1* in the W303a-related haploid W1588-4c, replacing it with the *NAT* gene (nourseothricin-resistance). This replacement was done by transforming W1588-4c with a PCR fragment generated by amplifying sequences of the plasmid pAG25 [2] using the primers V31539::DR F and W CAN1 KO R (listed in Table S8). Transformants were selected on YPD plates containing 0.1 mg/ml of nourseothricin. The resulting strain with this replacement is JSC10-1. The *CAN1* on chromosome V was deleted in the haploid PSL4 (isogenic with YJM789, genotype: *MATα ade2-1 ura3 gal2 ho::hisG*; [3]) by a similar procedure except that the PCR fragment used for transformation was produced by amplifying the *NAT* gene and surrounding sequences from JSC10-1 genomic DNA with primers CAN1-500 F and W2 CAN1 KO R. The resulting Nat^R^ transformant was JSC19-1.

A cassette of about 4 kb containing the *can1-100* gene and *KANMX* was made by inserting the geneticin-resistance gene, *KANMX*, near *can1-100* (between bases 31539 and 31540 on chromosome V) in W303a (Table S7). The *KANMX* sequences were amplified from the pFA6-kanMX4 plasmid [4] using the primers V31539::DR F and V31539::DR R. Transformants were selected on YPD medium containing 0.2 mg/ml of geneticin. The transformant with this cassette on chromosome V is JSC11-1. The *can1-100-KANMX* cassette was then amplified from JSC11-1 genomic DNA using the primers IV1510386::can1-100 F and IV1510386::can1-100 R, and transformed into JSC10-1. In the resulting transformant (JSC12-1), the *can1-100-KANMX* cassette is inserted onto chromosome IV between bases 1510386 and 1510387.

The *SUP4-o* gene was amplified using the YCPMP2 plasmid [5] as a template, and the primers YJMIV::Sup4-o F and YJMIV::Sup4-o R. The resulting PCR product was used to transform JSC19-1. Since JSC19-1 contains the ochre-suppressible *ade2-1* mutation, we selected Ade^+^ transformants. The resulting strain (JSC21-1) had an insertion of *SUP4-o* between bases 1510386 and 1510387 on chromosome IV.

The haploids JSC12-1 (isogenic with W303a except for changes introduced by transformation) and JSC21-1 (isogenic with YJM789 except for changes introduced by transformation) were mated, and the resulting diploid is JSC22-1. To prevent the diploid from undergoing sporulation, we replaced the *MATα* locus with the *HYG* gene. This replacement was performed using a PCR fragment generated by amplifying the pAG32 plasmid [2] using the primers MATALPHA NATF and MATALPHA NATR [3]. The resulting diploid JSC25-1 was used in our subsequent mapping experiments.

Although JSC25-1 was designed to allow us to select crossovers as Can^R^ red/white sectored colonies as we did on chromosome V, in this diploid, the suppression of the *can1-100* allele by the *SUP4-o* suppressor was inefficient. Thus, the diploid was not very sensitive to canavanine, resulting in high background growth of the diploid on plates containing canavanine, precluding the selection of canavanine-resistant colonies. Consequently, we screened for crossovers on chromosome IV by identifying red/white sectored colonies non-selectively as described in the main text.

As described in the Results section, we found that one of the recombination hotspots (HS4) mapped to the location of an inverted pair of Ty elements, near SGD coordinate 980 kb. The HS4 hotspot was specific to the W303a-derived chromosome. In order to test the hypothesis that these Ty elements formed a secondary DNA structure required for the hotspot activity, we created strains that had markers flanking the inverted repeat and various modifications of the inverted repeat structure. Since the conversion tracts associated with HS4 were very long (median length of 48 kb), we inserted the markers flanking the hotspot about 56 kb apart. The control strain with the intact HS4 hotspot was constructed in several steps. JSC52-1 is a W1588-4c derivative with *URA3* inserted centromere-distal to HS4 between SGD coordinates 1013217 and 1013218. The *URA3* gene in JSC52-1 was derived from the strain JAY291 (Table S7) by amplification of genomic DNA using the primers RE HS4 URA3 F and RE HS4 URA3 R. The Hyg^R^ haploid JSC54-1 was derived from the Ura^+^ transformant JSC52-1 by inserting the *HYG* gene at SGD coordinates 957578 to 957579. The fragment used for the transformation was generated by amplifying the plasmid pAG32 with primers RE HS4 HYG F and RE HS4 HYG R. The diploid JSC67-1 was generated by a cross of JSC54-1 to PSL4. The diploid JSC71-1 was derived from JSC67-1 by replacing the *MATα* gene with the *NAT* gene, using the same method described for the construction of JSC25-1. JSC71-1 was the control diploid for analysis of the strains that had alterations of HS4.

Three diploids with different modifications of HS4 were constructed. The diploid JSC73-2 strain had a deletion of the centromere-proximal Ty element, *YDRWTy2-3*. The first step in the construction of this strain was to replace the Ty element in JSC54-1 with the *KANMX* gene by transforming the strain with a PCR fragment generated by amplifying the pFA6-kanMX4 plasmid with primers HS4 Ty2-3::KAN F and HS4 Ty2-3::KAN R. The resulting Gen^R^ transformant is JSC57-1. The diploid JSC68-1 is the result of a cross of JSC57-1 with PSL4. JSC73-2 is derived from JSC68-1 by replacement of the *MATα* locus with the *NAT* gene.

In the diploid JSC74-1, the distance between the Ty elements of HS4 was expanded by insertion of the *KANMX* gene between SGD coordinates 987150 and 987225. The first step in the construction was to transform the haploid JSC54-1 with a *KANMX*-containing PCR fragment produced by amplifying the pFA6-kanMX4 plasmid with the primers HS4 Spacer::Kan F and HS4 Spacer::Kan R. The resulting Gen^R^ transformant was JSC59-2. The diploid JSC70-3 is a cross of JSC59-2 and PSL4, and JSC74-1 is a diploid with a deletion of *MATα* (performed as described above for strain JSC71-1).

In JSC77-1, the 5’ delta element of *YDRWTy2-3* was replaced with the *KANMX* gene. We first constructed a Gen^R^ derivative of JSC54-1 (JSC58-2) by transformation with a PCR fragment generated by amplification of the pFA6-kanMX plasmid with the primers HS4 Ty2-3::KAN F and HS4 DELTA19::KAN R. The diploid JSC75-1 is a cross of JSC58-2 and PSL4. The *MATα::NAT* derivative of JSC75-1 (JSC77-1) was constructed by transforming JSC75-1 with a PCR fragment generated by amplifying the pAG25 plasmid with the primers MATALPHA NATF and MATALPHA NATR [3].

#### Design of the SNP microarrays

The SNP microarrays that were used in our study were based on our own design and were generated by Agilent. The arrays contained about 9200 oligonucleotides, allowing us to analyze about 2300 SNPs. The majority of the oligonucleotides used to examine SNP-specific hybridization patterns were designed using the principles described by Gresham et al. [6] as employed in our previous study [7]. In addition, we incorporated information from SNP microarray experiments performed by D. R. Georgianna and J. H. McCusker (Duke University; personal communication) and data described by [8]. The sequences of the oligonucleotides used in construction of our SNP microarray are presented in Table S9.

**Supplemental Results and Discussion**

#### Conversion tracts associated with DSBs initiated during G1 or S/G2 of the cell cycle

As discussed in the main text, from the patterns of conversions associated with the crossover, we can infer whether the initiating DSB was generated in the chromosome before replication (G1) or during the S-/G2-phases of the cell cycle. For the crossovers associated with simple 3:1, 4:0, or hybrid 3:1/4:0 and 3:1/4:0/3:1 conversion events (approximately two-thirds of the total events), our conclusions about the timing of the DSB are fairly straightforward: simple 3:1 events are a consequence of S- or G2-initiated DSBs and 4:0 or simple hybrid tracts reflect G1-initiated DSBs. For most complex conversion events, however, the interpretation of the events as G1 or S/G2 can be more difficult. In this section, we discuss these more complex events.

The conversion tracts for all of the crossovers are depicted in Table S1. In this table, each sectored colony is represented by two lines, with the upper line showing the patterns of heterozygosity and homozygosity of SNPs in the red sector and the lower line indicating patterns of SNPs in the white sector. Green, red, and black line segments represent heterozygosity, homozygosity for W303a-derived SNPs, and homozygosity for the YJM789-derived SNPs, respectively. In Table S1, we divide all of the recombination events into five groups: A (crossovers without detectable conversions), B (simple 3:1 conversions), C (simple 4:0 conversions), D (simple 3:1/4:0 or 3:1/4:0/3:1 hybrid tracts), and E (complex conversion tracts). The Class A events cannot be characterized as G1- or S/G2-initiated, since they lack the diagnostic conversion tract. All other events were classified as G1- or S/G2-initiated as indicated by one or two asterisks, respectively, in Table S1.

One of the simplest indicators of a G1-initiated DSB is the presence of a 4:0 segment within a conversion tract [3, 9]. Although, in principle, these events could represent two independent 3:1 conversions, their frequency is much too high to represent two independent events [3]. Based on the criterion of a 4:0 conversion tract segment, all C and D, and most of the Class E events were initiated in G1.

There are a few conversion tracts classified as G1-initiated events that did not contain 4:0 regions. In Classes E8 and E9, there appears to be a crossover within a 3:1 conversion tract (Table S1). The pattern can be explained as a consequence of the conversion-associated repair of two broken chromatids with non-overlapping conversion tracts and a crossover between the converted segments (Fig. S2 in[3]). In 3:1 conversion events, the chromosome with the DSB is the recipient of information. For example, in Fig 1A, since the conversion event results in 3 copies of the W303a-derived SNPs (“red” SNPs) and one copy of the YJM789-derived SNPs (“black” SNPs), we infer that the conversion was initiated by a DSB on the YJM789-derived homolog. In addition, the region of the conversion tract that is homozygous for the W303a-derived SNPs is expected to be in the red sector and the heterozygous region of the tract is expected to be in the white sector (Fig 1A). Of the 34 3:1 conversion tracts observed in our study, 29 (Classes B1 and B2) had this pattern. In five tracts (Classes E3 and E4), the homozygous and heterozygous regions were in the “wrong” sectors. These patterns can be explained as a consequence of the repair of two broken chromatids (Fig. S1 in [3]). By similar explanations, Classes E5, E8, E9, E11, E12, and E31 are likely to reflect G1-initiated events.

There are alternative interpretations of the conversion events depicted in Table S1. For example, a 4:0 tract could reflect a DSB that was formed in G2 if the broken chromatid was segregated into the daughter cell without repair. If this broken chromosome was then replicated and repaired in G2 of the subsequent cycle, it would generate a 4:0 conversion. One argument against this scenario is that gamma-ray-treatment of G2-synchronized yeast cells results primarily in 3:1 events rather than 4:0 events [9]. It is also possible that some of the conversions classified as initiated in G2 are initiated in G1, if one of the DSBs was repaired associated with a very short heteroduplex tract. If the heteroduplex does not contain a SNP, it is undetectable as a conversion event. For this reason, our estimate of the fraction of G1 events is likely to be an underestimate. Despite these uncertainties, the most likely interpretation of our data is that both G1- and S-/G-initiated events contribute to the crossovers detected in our study.

#### Associations of gene conversion tracts with the chromosome elements shown in Table S4.

Based on many previous studies (such as [10]), the position of the DSBs that initiate mitotic recombination events are located within the boundaries of the gene conversion events. Therefore, we examined the frequencies of various chromosome elements (replication origins, palindromes, G4 quadruplex motifs, etc.; Table S4) within gene conversion tracts for their over- or under-representation compared to a random distribution. We analyzed the “windows” containing the gene conversion tracts (the sites of potential DSB initiation) using four different methods. For all four methods, the region included for the Class A events (crossover without a gene conversion event; Table S1) was between the heterozygous SNPs and the homozygous SNPs that most closely flanked the crossover.

For Method 1, we summed all conversion tracts of each event to generate the investigated “window.” The sequences included for each conversion tract were from the last heterozygous SNP to the first homozygous SNP representing the crossover. For example, for sectored colony 112 (Fig. S1), the conversion tract extended between coordinates 663372 and 700426 (Tables S1 and S2). For Method 2, we also summed the conversion tracts for each event, but excluded those events in which the conversion tract was greater than 20 kb. For Method 3, we restricted the “window” of the conversion event to that portion of the conversion event most likely to contain the DSB. More specifically, if the conversion event was a 3:1/4:0 tract, we used a window that was restricted to the 4:0 portion of the tract, since (Fig 1C) the 4:0 region of a hybrid tract is the expected location of the recombinogenic DSB. In complex conversion tracts with multiple 4:0 regions, all 4:0 regions were counted in the window for that sectored colony. In Table S1, the regions that were included in our analysis by Method 3 are outlined in blue rectangles. Method 4 was identical to Method 3, except we excluded those events in which the tract size was greater than 20 kb. The complex recombination events that had conversion events initiated on both homologs (Classes E29-E31) were not included in any of the calculations.

In addition to using different methods of analyzing the data, as described in the main text, we chose five different datasets to examine: 1) all of the data derived from JSC25 (“JSC25 all” in Table S5 and S6), 2) recombination events initiated in G1 (“JSC25 G1”), 3) events initiated in G2 (“JSC25 G2”), 4) events initiated on the W303a-derived homolog (“W303a all”), and 5) events initiated on the YJM789-derived homolog (“YJM789 all”). All five datasets were examined using all four methods. As an example of the analysis, we will describe the procedure to determine whether there was a significant enrichment of LTR (delta) elements associated with the gene conversion tracts in the dataset “JSC25 all” when examined by Method 1. The Saccharomyces Genome Database shows that there are 33 delta elements in the 1,061,175 bp region between *CEN4* and the *can1-100/KANMX/SUP-o* insertions. The sum of the gene conversions (calculated by Method 1) for the 135 (136 crossovers minus one double crossover) events examined in the “JSC25 all” dataset was 2,660,253 bp. Since the density of delta elements on the right arm of chromosome IV is 1 per 32,157 bp (1,061,175 divided by 33 delta elements), the expected number of delta elements in the conversion window of 2,660,253 bp is 83 (2,660,253 bp conversion window divided by 32,157 bp), and the expected number of delta elements not included in the conversion window is 4,372 (135 events times 33 LTRs per event minus the 83 LTRs expected within the conversion window). The observed number of delta elements in the 2,660,253 bp conversion window was 130 and the observed number of delta elements not included in the conversion window was 4,325 (135 events times 33 LTRs per event minus the 130 LTRs observed within the conversion window). We compared the numbers of expected (83 and 4,372) and observed (130 and 4,325) events by a chi-square goodness-of-fit test using the Microsoft Excel “chisq.test” function. The p value for this comparison was 1.5 x 10^-7^. Since we analyzed the dataset for 18 different chromosome elements (listed in Table S4), we determined that this p value was still significant after correcting for multiple comparisons [11].

A similar procedure to that described above was used for all of the other comparisons and those comparisons that showed a significant over-representation of elements are shown in Table S5; those comparisons that had a the significant under-representation of elements are shown in Table S6. The conversion window sizes (shown in kb in parentheses) used for the different methods (indicated by the number in boldface) and the various datasets were: **1** JSC25 all (2660253); **2** JSC25 all (774888); **3** JSC25 all (1765865); **4** JSC25 all (689319); **1** JSC25 G1 (2129610); **2** JSC25 G1 (489295); **3** JSC25 G1 (1244102); **4** JSC25 G1 (410034); **1** JSC25 G2 (310456); **2** JSC25 G2 (203353); **3** JSC25 G2 (292886); **4** JSC25 G2 (185983); **1** W303a all (1492068); **2** W303a all (397036); **3** W303a all (932621); **4** W303a all (336974); **1** YJM789 all (947998); **2** YJM789 all (295812); **3** YJM789 all (615629); **4** YJM789 all (270305).

For some of the elements examined, the size of the element was a significant fraction of the size of the conversion tract. If the size of the element was greater than 10% of the median size of the conversion tracts, we added a correction factor to the conversion tract window size that was equivalent to the number of conversion tracts multiplied by the size of the element. For example, the median size of the conversion tracts of the conversion events determined by Method 1 for all JSC25 events was 12241 bp. The size of Ty elements is 5921 bp, considerably greater than 10% of the median conversion tract size. The total number of conversion events examined by Method 1 for JSC25 was 135. Therefore, the corrected window used for the analysis of Ty1 elements was 2660253 bp (unnormalized window size) added to 135 x 5921 bp, summing to 3459588 bp. The reminder of the analysis was performed as described above for the unnormalized data.

*Analysis of conversion tract termination regions*.

In addition to looking for chromosome elements that were over- or under-represented within conversion tracts, we looked for elements that were over- or under-represented at the termini of the conversion tracts. For this analysis (which excludes simple crossovers), each conversion tract was divided into two windows, one for the first transition of the conversion and one for the last transition. For example, the first transition of the conversion tract associated with sector 1RW (Table S2) was represented by a window extending from 1248863 to 1248966; these coordinates flank the transition between heterozygous markers and homozygous markers in the white sector. The second window for the sector is between coordinates 1273350 and 1274404. These windows were examined for the presence of chromosome elements listed in Table S4 using the same procedures described above for our conversion tract analysis. Since the median size of the conversion tract termination windows was small (910 bp), for many of our comparisons, we normalized the size of the windows by the method described in the previous paragraph; any element that overlapped with these windows was counted as being present within the window.

By these calculations, the only chromosome elements that were significantly (p = 2 x 10^-7^) enriched at the termini of conversion tracts were LTR elements. There are 33 such elements on the right arm of chromosome IV, about half of which are associated with Ty1 or Ty2 sequences (Table S4). LTR elements that were not associated with Ty elements were not significantly over-represented at the termini of conversion tracts (p = 0.5). The right arm of the YJM789-derived chromosome IV is missing all or most of five of the eight Ty elements that are present on the W303a-derived homolog (*YDRCTy1-1*, *YDRWTy2-2*, *YDRCTy1-2*, *YDRWTy2-3*, and *YDRCTy1-3*). We found that the LTR events that flanked these heterozygous elements (*YDRCdelta7*, *YDRCdelta8*, *YDRWdelta13*, an unannotated delta element at the end of *YDRCTy1-2*, *YDRWdelta19* (981171-981502), and *YDRCdelta22*) were significantly (p = 3 x 10^-5^) over-represented at the ends of conversion tracts. The interpretation of this result is discussed in the main text. One element (tandem repeats) was significantly (p = 0.0001) under-represented.

#### Analysis of mitotic recombination hotspot activities.

Although the distribution of recombination events depicted in Fig 4A appear strikingly non-random, we did several tests to confirm this conclusion. One complication in the depiction of Fig 4 (as explained previously in the text) is that the frequency with which a SNP will be involved in a conversion event is affected by both the frequency of initiating DSBs that occur near the SNP and the size of the conversion tracts extending from the initiating DSB. To eliminate the effect of the size of the conversion event on our calculations, for the bin analysis shown in Fig S2, each conversion tract was represented by a single point/coordinate.

The positions of the points used in this analysis for each sectored colony are given in Table S3. For the majority of the conversion tracts, the point that represented the tract was at the center of the DSB region as determined for Methods 3 and 4 described above. For Class A events (crossovers without conversions), the event was represented by the midpoint between the last heterozygous SNP and the first homozygous SNP. For conversion tracts that were discontinuous (regions of conversion separated by regions of heterozygosity such as Classes E2 and E7), the tract was treated as a single conversion tract with the point located in the middle of the tract. The events in which both homologs were donors (Classes E30-E32) were not used for this analysis.

As described in the main text, we then distributed the “point” conversion events into bins of 200 kb on chromosome IV (Fig S2) and tested whether the distribution was random. The analysis was done for all of the data taken together (Fig S2A), and for the W303a-associated (G1-initiated and S-/G2-initiated) and YJM789-associated (G1-initiated and S-/G2-initiated) events treated separately (Fig S2B). By chi-square goodness-of-fit tests, we found a very significant hotspot for W303a-associated G1-initiated events in the bin with coordinates 845-1045 kb. In addition, by contingency chi-square analysis, we found a significant difference in hotspot activity between the homologs, and a significant difference between the distributions of G1- and S/G2-initiated events.

### *Complex conversion tracts*

The simple conversion events shown in Figure 1 can be simply explained as resulting from the repair of one (Fig 1A) or two (Fig 1B and 1C) DSBs by the canonical DSB repair model [3, 7, 12]. In this model, DSB formation is followed by processing of the broken ends to yield ends with 3’ overhangs. These single-stranded DNA molecules “invade” the homolog forming regions of heteroduplex. Gene conversion events reflect repair of mismatches within the heteroduplex; repair of mismatches during meiotic conversion events is usually continuous, with mismatches deleted from one strand and corrected using the other strand as a template [13].

About one-third of the conversion tracts had more complex patterns that require modifications of the canonical model. For example, in Figure S7, a 3:1 conversion tract is interrupted by a region that is heterozygous in both sectors. This pattern is consistent with repair of a DSB formed during S or G2, followed by heteroduplex formation and “patchy” repair of mismatches. Since many of the classes of complex conversion events are similar or identical to complex tracts that we observed previously in other studies [7], we will not discuss models for their formation in any detail.

## References

1. Barbera, M.A. and T.D. Petes (2006) Selection and analysis of spontaneous reciprocal mitotic cross-overs in *Saccharomyces cerevisiae*. P Natl Acad Sci USA 103: 12819-24.

2. Goldstein, A.L. and J.H. McCusker (1999) Three new dominant drug resistance cassettes for gene disruption in *Saccharomyces cerevisiae*. Yeast 15: 1541-53.

3. Lee, P.S., P.W. Greenwell, M. Dominska, M. Gawel, M. Hamilton, et al. (2009) A fine-structure map of spontaneous mitotic crossovers in the yeast *Saccharomyces cerevisiae*. PLOS Genet 5: e1000410.

4. Wach, A., A. Brachat, R. Pohlmann, and P. Philippsen (1994) New heterologous modules for classical or PCR-based gene disruptions in *Saccharomyces cerevisiae*. Yeast 10: 1793-808.

5. Pierce, M.K., C.N. Giroux, and B.A. Kunz (1987) Development of a yeast system to assay mutational specificity. Mutat Res 182: 65-74.

6. Gresham, D., B. Curry, A. Ward, D.B. Gordon, L. Brizuela, et al. (2010) Optimized detection of sequence variation in heterozygous genomes using DNA microarrays with isothermal-melting probes. P Natl Acad Sci USA 107: 1482-7.

7. St Charles, J., E. Hazkani-Covo, Y. Yin, S.L. Andersen, F.S. Dietrich, et al. (2012) High-resolution genome-wide analysis of irradiated (UV and gamma-rays) diploid yeast cells reveals a high frequency of genomic loss of heterozygosity (LOH) events. Genetics 190: 1267-84.

8. Winzeler, E.A., C.I. Castillo-Davis, G. Oshiro, D. Liang, D.R. Richards, et al. (2003) Genetic diversity in yeast assessed with whole-genome oligonucleotide arrays. Genetics 163: 79-89.

9. Lee, P.S. and T.D. Petes (2010) Mitotic gene conversion events induced in G1-synchronized yeast cells by gamma rays are similar to spontaneous conversion events. P Natl Acad Sci USA 107: 7383-8.

10. Tang, W., M. Dominska, P.W. Greenwell, Z. Harvanek, K.S. Lobachev, et al. (2011) Friedreich's ataxia (GAA)n*(TTC)n repeats strongly stimulate mitotic crossovers in *Saccharomyces cerevisae*. PLOS Genet 7: e1001270.

11. Hochberg, Y. and Y. Benjamini (1990) More powerful procedures for multiple significance testing. Stat Med 9: 811-8.

12. Szostak, J.W., T.L. Orr-Weaver, R.J. Rothstein, and F.W. Stahl (1983) The double-strand-break repair model for recombination. Cell 33: 25-35.

13. Petes, T.D., R.E. Malone, and L.S. Symington (1991) Recombination in Yeast. In: Broach J.R. , E.W. Jones, and J.R. Pringle, editors. The Molecular and Cellular Biology of the Yeast *Saccharomyces*. Cold Spring Harbor Press: Cold Spring Harbor. pp. 407-521.

14. Mancera, E., R. Bourgon, A. Brozzi, W. Huber, and L.M. Steinmetz (2008) High-resolution mapping of meiotic crossovers and non-crossovers in yeast. Nature 454: 479-85.

15. Lisnic, B., I.K. Svetec, H. Saric, I. Nikolic, and Z. Zgaga (2005) Palindrome content of the yeast *Saccharomyces cerevisiae* genome. Curr Genet 47: 289-97.

16. Gelfand, Y., A. Rodriguez, and G. Benson (2007) TRDB--the Tandem Repeats Database. Nucleic Acids Res 35: D80-7.

17. Capra, J.A., K. Paeschke, M. Singh, and V.A. Zakian (2010) G-quadruplex DNA sequences are evolutionarily conserved and associated with distinct genomic features in *Saccharomyces cerevisiae*. PLOS Comput Biol 6: e1000861.

18. Fachinetti, D., R. Bermejo, A. Cocito, S. Minardi, Y. Katou, et al. (2010) Replication termination at eukaryotic chromosomes is mediated by Top2 and occurs at genomic loci containing pausing elements. Mol Cell 39: 595-605.

19. Nagalakshmi, U., Z. Wang, K. Waern, C. Shou, D. Raha, et al. (2008) The transcriptional landscape of the yeast genome defined by RNA sequencing. Science 320: 1344-9.

20. Szilard, R.K., P.E. Jacques, L. Laramée, B. Cheng, S. Galicia, et al. (2010) Systematic identification of fragile sites via genome-wide location analysis of gamma-H2AX. Nat Struct Mol Biol 17: 299-305.

21. Azvolinsky, A., P.G. Giresi, J.D. Lieb, and V.A. Zakian (2009) Highly transcribed RNA polymerase II genes are impediments to replication fork progression in *Saccharomyces cerevisiae*. Mol Cell 34: 722-34.

22. Thomas, B.J. and R. Rothstein (1989) Elevated recombination rates in transcriptionally active DNA. Cell 56: 619-30.

23. Zhao, X., E.G. Muller, and R. Rothstein (1998) A suppressor of two essential checkpoint genes identifies a novel protein that negatively affects dNTP pools. Mol Cell 2: 329-40.

24. Argueso, J.L., M.F. Carazzolle, P.A. Mieczkowski, F.M. Duarte, O.V. Netto, et al. (2009) Genome structure of a *Saccharomyces cerevisiae* strain widely used in bioethanol production. Genome Res 19: 2258-70.
